# Object-centered sensorimotor bias of torque control in the chronic stage following stroke

**DOI:** 10.1038/s41598-022-18754-z

**Published:** 2022-08-25

**Authors:** Thomas Rudolf Schneider, Joachim Hermsdörfer

**Affiliations:** 1 Chair of Human Movement Science, Department of Sport and Health Sciences, Technical University of Munich, Georg-Brauchle-Ring 60/62, 80992 Munich, Germany; 2 Department of Neurology, Cantonal Hospital of St. Gallen, Rorschacher Str. 95, 9007 St. Gallen, Switzerland

**Keywords:** Neuroscience, Neurology

## Abstract

When lifting objects whose center of mass (CoM) are not centered below the handle one must compensate for arising external torques already at lift-off to avoid object tilt. Previous studies showed that finger force scaling during object lifting may be impaired at both hands following stroke. However, torque control in object manipulation has not yet been studied in patients with stroke. In this pilot study, thirteen patients with chronic stage left hemispheric stroke (SL), nine patients with right hemispheric stroke (SR) and hand-matched controls had to grasp and lift an object with the fingertips of their ipsilesional hand at a handle while preventing object tilt. Object CoM and therewith the external torque was varied by either relocating a covert weight or the handle. The compensatory torque at lift-off (Tcom) is the sum of the torque resulting from (1) grip force being produced at different vertical finger positions (∆CoP × GF) and (2) different vertical load forces on both sides of the handle (∆Fy × w/2). When having to rely on sensorimotor memories, ∆CoP × GF was elevated when the object CoM was on the ipsilesional-, but decreased when CoM was on the contralesional side in SL, whereas ∆Fy × w/2 was biased in the opposite direction, resulting in normal Tcom. SR patients applied a smaller ∆CoP × GF when the CoM was on the contralesional side. Torques were not altered when geometric cues were available. Our findings provide evidence for an object-centered spatial bias of manual sensorimotor torque control with the ipsilesional hand following stroke reminiscent of premotor neglect. Both intact finger force-to-position coordination and visuomotor control may compensate for the spatial sensorimotor bias in most stroke patients. Future studies will have to confirm the found bias and evaluate the association with premotor neglect.

## Introduction

Many stroke survivors suffer from impairments of dexterous upper-limb function affecting their functional independence as well as quality of life^1–3^. Weakness, spasticity and a loss of selective finger movements of the contralesional upper extremity consequent to lesions of the primary cortex or the corticospinal tract as well as impaired manual dexterity due to somatosensory deficits linked to thalamic or parietal cortical lesions are clinically well recognized consequences of stroke and have been the focus of physical rehabilitation research as they contribute most to functional impairments [for review see^2^]. Consequently, stroke survivors with contralateral hemiparesis must rely on their ipsilesional, i.e. non-paretic, hand to a great extent to perform activities of daily living. However, a growing number of studies demonstrates that fine motor performance of the ipsilesional upper limb is also substantially deprived following stroke^4,5^. Impaired fine motor control of the ipsilesional hand is evident in clinical motor function tests like the Jebsen Hand Function Test^5–10^, in finger-tapping^11,12^, and tests of fine motor dexterity, e.g. the 9-hole-peg test^8,9,13–15^. Subtle losses in dexterity of the ipsilesional hand are relevant for performance in activities of daily living and thus threaten the regaining of functional independence following stroke. Accordingly, poorer performance with the ipsilesional hand was confirmed in activities of daily living like the one-handed binding of shoes^16^ and the preparation of meals^17^. Recent research highlights that ipsilesional hand performance is highly relevant for the functional independence following left hemisphere stroke^18^. Therefore, identifying the factors underlying impaired, ipsilesional upper limb control and developing targeted rehabilitation regimes is of paramount importance.

Kinematic analyses of reaching tasks revealed that ipsilesional motor deficits are hemisphere dependent and reflect lateralization of motor function. Movements of the ipsilesional arm are slower and more variable following left hemisphere damage while final position accuracy is decreased after right hemisphere damage^18–24^. These observations led to the proposal of a “dynamic dominance” hypothesis of motor lateralization stating that the dominant hemisphere is specialized for the coordination of limb and task dynamics, i.e. movement trajectories, while the nondominant hemisphere is responsible for achieving the final, i.e. steady-state, end-effector positions and stabilizing external loads^22,25–27^. Ipsilesional motor deficits in reaching tasks scale with the severity of contralesional arm impairment, i.e. the more severe the contralesional arm paresis, the larger the ipsilesional motor deficits^14^, and correlate with apraxia scores in patients with left-hemispheric stroke^7,8^ although the relationship may be complex^21^.

One elegant way to study complementary pathophysiologic aspects of manual dexterity following stroke is to examine kinetics, i.e. forces and torques, when patients execute elementary grasp-to-lift tasks. In healthy adults grip forces (GF), i.e. the force acting orthogonal to the grip surface, and load forces (LF), i.e. the forces directed tangentially upwards, rise in parallel and are precisely scaled to the anticipated characteristics of both the object (weight, frictional characteristics) according to previous experience, i.e. sensorimotor memories^28,29^, and visual object characteristics, e.g. size, material, arbitrary cues, object identity^30–35^, and the dynamics of the task [for review see:^36^].

Hemiparetic patients with stroke typically exert increased grip forces when lifting objects with their more affected, contralesional, hand which can be partially attributed to disturbed sensorimotor integration^37–43^. Moreover, studies investigating the ipsilesional, non-paretic, hand of stroke survivors also found elevated grip force levels^44–46^ as well as an increased grip force variability^45^ and disturbed anticipatory grip-to-load force coupling^46^. In contrast, gross grip strength is not reduced in the ipsilesional hand following stroke^5,9,10^. Adding to these problems in the task execution, the anticipatory planning of forces is also impaired following stroke. While the anticipatory scaling of grip forces according to object size is intact in stroke patients (Li et al.^63^), patients with left hemisphere damage failed to scale grip forces to the actual weight of objects of daily life when grasping and lifting them with their ipsilesional hand^47^. This GF scaling deficit was associated with scores of apraxia. Similarly, patients with left-sided middle-cerebral artery (MCA) stroke could not use color-cues associated with object weight to scale grip forces with either hand, whereas patients with right MCA stroke only showed impaired force scaling with their contralesional hand^48^.

The control of torques when lifting an object with an eccentric center of mass (CoM) relative to the hand is another essential aspect of dexterous object handling in daily life which has been extensively studied in healthy adults over the last two decades. To prevent object tilt, e.g. when lifting a cup of tea at the handle, arising torques must be already compensated at the moment of object lift-off, i.e. before full sensory feedback of object toque is available. Two torque components add up to the total torque applied by the fingers in the direction of interest. These are a) the product of the load force difference between grasp-sides (∆Fy) and half the grasp-width (w/2) and b) the product of the distance between the finger centers of pressure on the grasp surfaces (∆CoP) and the grip force (GF). Therefore, the digit placements and grip- and load forces must be coordinated to apply adequate counteracting torques at lift-off [for review see^49^]. Healthy adults learn to modulate both their digit centers of pressure and digit forces by placing the digit(s) on the side of the center of mass higher and applying more load force at the digit on that side according to previous experience^50–52^, even when object dynamics change unpredictably^53,54^. Furthermore, subjects can visually process salient object shape/geometry cues to infer the weight distribution of the object and plan torques accordingly^51,55–57^. To generate adequate compensatory torques, digit -forces and -placements are covaried by a high-, respectively task-level control. This principle of force-to position covariation is grasp-type independent^58^ and was shown for grasps with a precision grip^50^, tripod grip^59^, whole hand grasps^60^ as well as for bimanual grasps^61^. Although torques can be applied by any combination of digit center of pressure differences between the grasp sides (∆CoP) and load force partitioning between sides (∆Fy) as long as the resulting torque components add up to the required total torques, we recently demonstrated that an adequate finger-tip positioning and a predominant torque exertion by the product of ∆CoP and GF is essential for a force efficient task execution^62^. Whether these aspects of high-level torque control are impaired at the ipsilesional hand of patients with unilateral stroke has not been investigated, yet.

In the present study, we examined whether the anticipatory torque control with the ipsilesional hand when lifting an object with a varying asymmetric weight distribution is impaired in the chronic stage following unilateral stroke. We tested two cue conditions. The first was a ‘no-cues’ condition in which the position of a covert weight was changed while object shape (inverted T) was not informative of the CoM. In this condition, subjects had to rely on sensorimotor memories from the last lift or lifts. In the second condition the visually salient object geometry was congruent with weight distribution (L-shape) allowing visual inference of CoM. Moreover, two sequence conditions, one in which the mass distribution was constant over a block of trials and one in which it could change from trial to trial were employed for both cue conditions.

Since the right-hemisphere is proposed to be responsible for end-effector positions according to the “dynamic dominance” model^25^ we expected that patients with right hemisphere damage would fail to learn to position their fingers for an adequate torque component ∆CoP × GF, but would correct for this by compensatory ∆Fy (× w/2) resulting in successful total torque compensation.

Based on the hypothesized role of the left hemisphere in the dynamic phase of an action, we hypothesized a less accurate coordination of fingertip load forces (∆Fy) to the present ∆CoP and consequently less successful predictive torque compensation in patients with left-hemispheric-, but not right hemispheric stroke, irrespective of the side of the object center of mass (CoM). Moreover, we presumed that patients with signs of apraxia would present an accentuated impairment of force-to position coordination and consequently torque compensation.

However, as stroke patients previously exhibited mostly intact visuomotor processing of size and weight cues to scale finger-tip forces^63,64^ we expected that most stroke patient can improve torque anticipation when salient-geometric cues are provided. As an exception, we presumed that patients with hemispatial neglect might fail to utilize a lateralized geometric cue indicating a CoM on the contralesional side.

Concerning grip force levels, we expected to observe elevated and more variable GF levels in both stroke groups based on previous studies^44,45^.

## Materials and Methods

### Participants

Overall, 13 patients with chronic-stage left hemispheric stroke (SL group: 6 female, mean age 63.3 ± 16.3 years, mean years since onset of stroke (YOS): 6.06 ± 4.10 years) and 9 patients with chronic-stage right hemispheric stroke (SR group: 5 female, mean age 63.9 ± 6.7 years, mean YOS 7.5 ± 5.7 years) were tested with their ipsilesional hand. 15 healthy adults who conducted the experiment with their left hand (CL group: 6 female, mean age 63.0 ± 13.1 years) and 9 healthy adults who conducted the experiment with their right hand (CR group: 4 female, mean age 69.8 ± 3.8 years) served as control groups. Patients with a single unilateral cerebrovascular event older than 6 months and no evidence of bilateral lesions in their medical reports were recruited from the community with the help of physiotherapists, occupational therapists, speech therapists and neuropsychologist in the greater Munich area (see Acknowledgements). All participants reported to be right handed.

Table 1 provides group summaries of the demographic and clinical characteristics as well as the results of the performed neglect and apraxia tests together with the statistical results of between group tests (ANOVA, respectively t- tests for numerical data, chi-square tests for categorical data). Individual patient’s data are outlined in Supplementary Table S1.

**Table 1:** Group summary of the demographics, clinical data, the coefficients of fraction, maximum voluntary GF, the results of the pantomime and imitation tests of G. Goldenberg (see also^66,71,91^ as well as the results of the line bisection test, letter cancellation test^73^, and a Posner type reaction time test^76^.

	CL (*N* = 15)	CR (*N* = 9)	SL (*N* = 13)	SR (*N* = 9)	*p* value
**Age**					0.564^1^
Mean (SD)	63.0 (13.1)	69.8 (3.8)	63.3 (16.7)	63.8 (6.7)	
Range	24.9–80.5	65.3–76.3	24.5–79.8	50.4–72.2	
**Gender**					0.907^2^
m	9 (60.0%)	5 (55.6%)	7 (53.8%)	4 (44.4%)	
f	6 (40.0%)	4 (44.4%)	6 (46.2%)	5 (55.6%)	
**Stroke Type**					0.042^2^
i	0	0	10 (76.9%)	5 (55.6%)	
h	0	0	0 (0.0%)	4 (44.4%)	
i, h	0	0	1 (7.7%)	0 (0.0%)	
h, i	0	0	2 (15.4%)	0 (0.0%)	
**Subcort./cort. lesion**					0.290^2^
sc	0	0	3 (23.1%)	4 (44.4%)	
sc, c	0	0	10 (76.9%)	5 (55.6%)	
**mRS**					0.702^1^
Mean (SD)	NA	NA	2.4 (0.8)	2.2 (1.2)	
Range	NA	NA	1.0–4.0	1.0–4.0	
**Years since stroke onset**					0.505^1^
Mean (SD)	NA	NA	6.1 (4.1)	7.5 (5.7)	
Range	NA	NA	1.1–15.0	2.2–19.9	
**Coefficient of static friction**					0.292^1^
Mean (SD)	0.9 (0.2)	1.0 (0.2)	0.9 (0.1)	0.9 (0.1)	
Range	0.6–1.2	0.7–1.2	0.7–1.1	0.7–1.0	
**Peak voluntary GF [N]**					0.609^1^
Mean (SD)	68.7 (24.1)	57.5 (17.7)	67.6 (20.7)	66.0 (16.0)	
Range	34.1–111.1	35.4–93.1	34.7–98.5	37.8–93.3	
**Imitation Hand**					0.070^1^
N	0	0	13	8	
Mean (SD)	NA	NA	18.7 (1.7)	19.9 (0.4)	
Range	NA	NA	15.0–20.0	19.0–20.0	
**Imitation Finger**					0.616^1^
Mean (SD)	NA	NA	18.6 (2.7)	19.1 (1.0)	
Range	NA	NA	11.0–20.0	17.0–20.0	
**Pantomime correct items [/20]**					0.224^1^
N	0	0	13	9	
Mean (SD)	NA	NA	17.2 (4.9)	19.3 (1.0)	
Range	NA	NA	3.0–20.0	18.0–20.0	
**Pantomime Score [/55]**					0.241^1^
Mean (SD)	NA	NA	50.2 (10.1)	54.3 (1.0)	
Range	NA	NA	19.0–55.0	53.0–55.0	
**Bisection Test: mean horizontal deviation [mm]**					0.432^1^
N	0	0	10	6	
Mean (SD)	NA	NA	− 0.3 (2.7)	1.0 (3.8)	
Range	NA	NA	− 4.9–4.5	− 3.8–6.6	
**Letter cancellation test: center of cancellation**					0.143^1^
N	0	0	11	5	
Mean (SD)	NA	NA	0.0 (0.0)	0.0 (0.0)	
Range	NA	NA	0.0–0.0	0.0–0.1	
**Letter cancellation test: overall letters found [/60]**					0.051^1^
Mean (SD)	NA	NA	59.1 (1.4)	57.2 (2.2)	
Range	NA	NA	57.0–60.0	54.0–60.0	
**Posner test: median reaction time [ms]**					0.346^1^
N	0	0	12	5	
Mean (SD)	NA	NA	557.3 (179.4)	474.4 (87.1)	
Range	NA	NA	324.0–960.5	403.5–617.0	
**Posner test: relative L-R reaction time difference [%]**					0.001^1^
Mean (SD)	NA	NA	− 9.1 (8.3)	14.6 (16.5)	
Range	NA	NA	− 20.4–9.6	0.3–39.7	

^1^Linear Model ANOVA, ^2^ Pearson’s Chi-squared test.

The *p*-values of between groups differences were based on ANOVA tests for numerical data (respectively t-tests if data were only obtained for the stroke groups) and on chi-square tests for categorical data. Abbreviations: Stroke type: i = ischemic; h = hemorrhagic; i, h = ischemic stroke followed by hemorrhage; h, i: hemorrhage with subsequent ischemic infarction. Subcort./cort. Lesion: purely subcortical (sc) or subcortical and cortical lesions (sc, c).

The experimental procedures were approved by the Institutional Review Board of the School of Medicine at the Technical University of Munich and were in accordance with the Declaration of Helsinki. All subjects were naïve to the purpose of the study and gave informed consent to participate in the study and have us collect relevant medical reports from their family doctor. Measurements took place at our lab as well as in patients’ homes between September 2016 and April 2017. All participants received 20 € for their participation in the study which lasted ~ 2 h.

### Modified rankin scale (mRS)

The modified Rankin Scale (mRS) was assessed as measure of the degree of disability or dependence in the daily activities using the simplified questionnaire proposed by Bruno et al.^65^.

### Apraxia tests

We administered two established tests of apraxia and video-recorded them for later analysis. Firstly, we examined the imitation of meaningless gestures of hand- and finger postures with the ipsilesional hand. Imitation scores below 18 of 20 for hand- and 17 of 20 for finger-postures were considered as suggestive of apraxia^66–69^. In addition to imitation, we examined pantomime of tool-use. Here, we showed patients pictures of one of 20 tools or objects of the daily life and asked them to mime specific action as if they were holding the object in their ipsilesional hand. We scored whether hand positions and movements were correct. Scores below 45/55 were considered as suggestive of apraxia^68,70,71^.

### Tests of visual hemispatial neglect

The presence of hemispatial neglect was assessed by the (a) line bisection-test in which a deviation of more than 6 mm from the midpoint indicates hemispatial neglect^72^, (b) the letter cancellation test with performance quantified by the center of calculation (CoC) score introduced by Rorden and Karnath^73^—i.e. an absolute CoC score above 0.083 indicates presence of hemispatial neglect—and (c) a Posner type spatial cueing test^74^ implemented in the free computer test battery PEBL [version 0.14,^75^]. In the latter, patients sat in front of a 15.6-inch Lenovo laptop. After a cue to the left, right or both sides (neutral) was provided, indicating where the response is likely to be, patients had to press a key when they detected a stimulus either to the left or right of fixation. As measure of a hemispatial visual bias we calculated the standardized median reaction time difference between trials with stimuli to the left and to the right of fixation (overall 200 trials, 100 trials per stimulus side, cues were valid in 120 trials, neutral in 40 trials, and invalid in 40 trials). Reaction time differences between stimuli on the left and right side in Posner-type reaction time tests were shown to be more sensitive than paper and pencil based tests in detecting hemispatial neglect^76^. However, there is no established cut-off defining hemispatial neglect.

### Experimental design and statistical analyses

#### Apparatus

**Figure 1:**
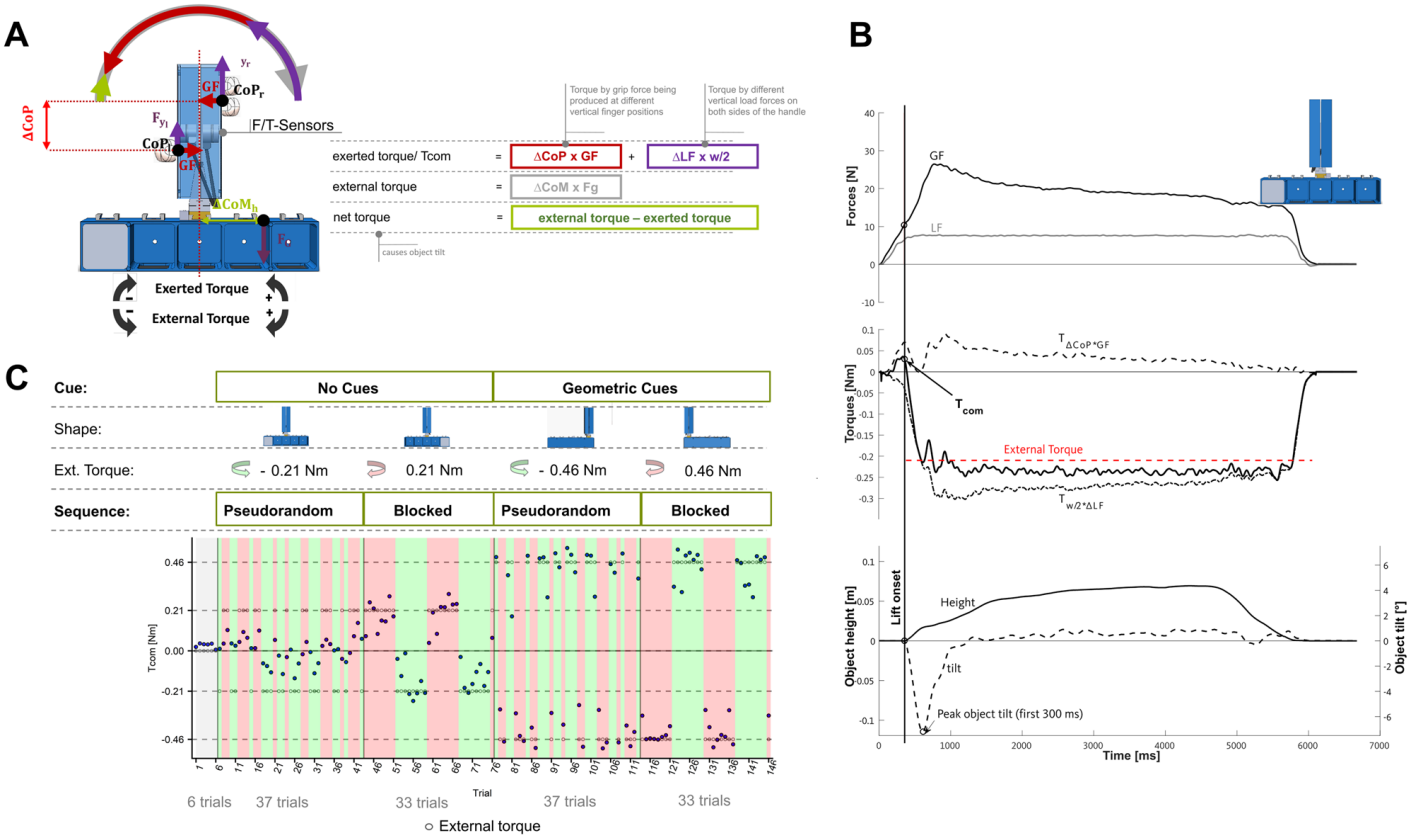
Experimental apparatus, variables and design. (**A**) The custom-built grip-device consists of a handle element mounted centrally on a horizontal bar (frontal view). The handle element allowed subjects to freely choose digit placement on the grip surfaces (40 × 120 mm) covered with sandpaper. Two 6-axis-force/torque sensors were mounted under the grasp surfaces. In the ‘no cues’ condition a hidden weight was either placed in the left or right cavity resulting in an external torque after lift-off. The exerted total torque is the sum of the torque components ∆CoP × GF and ∆Fy × w/2 and must compensate for the external torque to prevent object tilt. (**B**) The recorded experimental variables are illustrated for an exemplary trial, the torque variables at lift off were considered to be indicators of anticipatory torque control. (**C**) The experimental protocol comprised the two cue- conditions ‘no cues’ in which the center of mass (CoM) was changed by placing a hidden weight either on the left or the right (with the handle being positioned above the middle cavity), resulting in external torque of ± 0.21 Nm after liftoff, and the ‘geometric cues’ condition in which the handle was either mounted above the left or right cavity (with the hidden weight inserted in the central cavity) , resulting in external torque of ± 0.46 Nm after liftoff. The order of the conditions and first CoM side was randomly assigned to participants (see Supplementary Table 1). For each cue-conditions participants first completed a pseudorandom sequence of 37 trials in which the CoM could change from trial to trial and 33 trials in which the CoM stayed constant for 8 trials before it was inverted.

Subjects were instructed to reach, grasp, lift and replace a custom made, grip device with the thumb opposing the index and the middle finger^53^ (see Fig. 1A). The grasp surfaces (120 × 40 mm) were covered with fine-grained sand paper (2000 grit). Two 6-axis force/torque-sensors (ATI Nano-17 SI-50–0.5, ATI Industrial Automation; force range: 50,50, and 70 N for x-, y-, and z-axes, respectively; force resolution: 0.012 N; torque range 0.5 Nm; torque resolution: 0.063 Nmm, sampling rate 200 Hz) recorded the forces and torques applied on both grasp sides. Position and orientation data of the device were measured by a lightweight magnetic position/orientation-tracker (TrakSTAR, Ascension Technology Corporation, accuracy: 1.4 mm RMS, 0.5 degrees RMS, sampling rate 200 Hz) fixed on top of the horizontal base. Data collection was synchronized using custom software written in Matlab 2016a (MATLAB, RRID:SCR_001622). Both the position of the handle device on top of the base as well as the location of a 250 g aluminum weight which was put into cavities of the base hidden by a lid could be altered to vary the object’s center of mass (CoM) relative to the hand (see Experimental Protocol).

#### Determining the static coefficient of friction, μ_s_, at slip onset

Prior to the main experiment, subjects were asked to lift and hold the grip device in a three-finger precision grip with the thumb, index and middle fingers of the hand used for the upcoming lifting task and slowly release it until the object slipped. We estimated the average static friction coefficient, μ_s_, at the digit—surface contacts, by calculating the ratio between the load- and grip force at slip onset which was visually detected by a sudden drop in the load force and height. Overall, μ_s_ could be successfully calculated in 121 slip-trials. The averaged μ_s_ estimates are based on an average of 2.63 slip-trials per subject (SD 0.77, median 3, range 1–4).

#### Maximum GF

Prior to the main experiment we had participants pinch the grasp surfaces as hard as they could in the specified three-finger precision grip twice for five seconds and determined the highest applied GF as maximum GF.

#### Experimental task

For the main experiment, we instructed participants to start reaching for the grasp-device after a signal tone, grasp the grasp surfaces with the fingertips of the thumb-, index- and middle finger in a precision grip, lift it in a smooth movement to a height of ~ 5–10 cm while minimizing object tilts and hold the object steady thereafter. A second tone 4 s after the first signaled subjects to replace the device. Patients were allowed to position and orient the object on the table in a way that allowed for a comfortable wrist position for grasping.

#### Experimental protocol

First, participants conducted six practice grasp-to-lift trials in which the object’s CoM was below the middle of the handle (zero external torque).

Subsequently, the main experimental protocol contained two sequence conditions and two cue conditions (see Fig. 1C). In the ‘no cues condition’, the object handle was attached over the center of the base (symmetric, inverted T-shape) and the center of mass was varied by placing a covert 250 g aluminum weight into either the outer left or outer right hidden cavity of the horizontal base, resulting in external torques of ± 0.21 Nm (see Fig. 1C). In the ‘geometric cues condition’, in contrast, the aluminum weight was constantly placed in the center cavity, but the handle was either positioned on top of the left or right object edge creating an asymmetric L-shape and resulting in an external torques of ± 0.46 Nm (see Fig. 1C). As convention, negative signs denote a counter-clockwise external torque. The total object weight was 750 g.

In both cue-conditions, participants first conducted 37 trials in the ‘pseudorandom’ sequence-condition in which the CoM was changed in a pseudo random fashion which could not be predicted by the participants (see Fig. 1C). Participants had to close their eyes while the hidden weight was removed and placed back either into the same or the opposite position after each trial.

This was followed by the blocked sequence-condition in which the CoM remained constant for 8 trials per block before the CoM changed side for the next blocks. Participants were informed about the CoM change between blocks but were restricted of watching the configuration change. The blocked-sequence encompassed 4 complete blocks and the first trial of the 5th block, i.e. 33 trials. The succession of the pseudorandom and blocked sequence-condition trials was performed for both the no-cues and geometric-cues conditions, amounting to a total of 140 main trials per participant. We randomly assigned the order of the two cue conditions and the initial CoM side for the first trial for the no-cues- and geometric-cues conditions to the participants.

#### Data processing

Data were processed and analyzed with custom software written in Matlab 2016a. The collected force/torque data was filtered through a sixth-order Butterworth low-pass filter with a cutoff frequency of 14 Hz. The index and middle finger contacting the same grip side produced net mechanical forces and moments equivalent to the sum of their individual actions and were hence considered as a virtual finger^77^. We analyzed the exerted total torque (Tcom) as well as the torque components ΔFy*w/2 and ΔCoP *GF outlined below as well as the grip force (GF) at the moment of object lift off, defined as the moment 10 ms prior to which the vertical position of the object raised above a threshold of 0.2 mm.

We examined the following experimental variables (see Fig. 1B):

1. Grip force (GF) was defined as the mean normal force directed orthogonal towards the grip surfaces.
2. ∆CoP at lift-off was defined as the vertical difference between the center of pressure (CoP) on the right and the left grip sides at the moment of lift-off.
3. Tcom, the compensatory torque exerted at object lift off, is an established indicator of torque anticipation^50,78,79^. Tcom is the sum of: (a) ΔCoP *GF, the product of GF and ΔCoP and b) ΔFy*w/2, the torque generated by the product of the difference between the right and left load force and half the distance between the grip-surfaces $\left( {\frac{{\text{w}}}{2} = \,{2}0.{4} {\text{mm}}} \right)$. With the chosen sign conventions, Tcom matches in sign with the external torque when it counterbalances the exerted torque, e.g. is directed in opposing direction to the external torque. Hence, clockwise exerted torques were defined as negative and counter-clockwise torques as positive (see Fig. 1A and the supplementary material of^53^: 10.6084/m9.figshare.7683707). As outcome measures in the statistical analyses, we calculated the respective ratios between the torque variables and the external torque to compensate for, i.e.: $\frac{{{\text{Tcom}}}}{{{\text{External}}\,\,{\text{Torque}}}}$, $\frac{{\Delta {\text{Fy}}*{\text{w}}/2}}{{{\text{External}}\,{\text{Torque}}}}$ and $\frac{{\Delta {\text{CoP}}*{\text{GF}}}}{{{\text{External}}\,\,{\text{Torque}}}}$. This allows for direct evaluation of the success of torque anticipation as a ratio of 1 indicates perfect torque compensation and negative ratios indicate torques directed in the wrong direction. $\frac{{{\text{Tcom}}}}{{{\text{External}}\,{\text{Torque}}}}$ is the primary outcome variable, $\frac{{{{\Delta {\text{Fy}}* {\text{w}}}}/2}}{{\text{External Torque}}}$, $\frac{{{{\Delta {\text{CoP}} *{\text{GF}}}}}}{{\text{External Torque}}}$ are the secondary outcome variables, and ∆CoP and GF represent exploratory tertiary outcome variables.
4. Additionally, we estimated the average static coefficients of friction, $\mu{_{{\mathbf{s}}}}$, of each participant by averaging the ratios between the load force and grip force at the moment at which slips occurred in the slip-task to control for possible friction differences between groups.

#### Data management

Due to technical errors 1.58% (106/6716) of the measurements had to be discarded. We obtained 121 $\mu{_{{\mathbf{s}}}}$ estimates employing the slip-method.

#### Statistical analysis

Statistical analyses were performed in the R environment for statistical computing (version 4.0.3,^80^, R Project for Statistical Computing, (RRID):SCR_001905). To compare the demographic and clinical characteristic of the control- and stroke groups exploratory analyses of variance (ANOVA) tests for numerical data (respectively t-tests if data were only obtained for the stroke groups) and chi-square tests for categorical data were conducted as implemented in the ‘arsenal’ package^81^ (see Table 1).

We fitted separate linear mixed effects models (LMM) with random-intercepts estimating the random variance across subjects using the restricted maximum likelihood criterion as implemented in the ‘lme4’-^82^ package for the dependent primary and secondary outcome variables and every experimental condition.

The fixed effect predictors of the models for the blocked sequence condition were: the participant ‘group’, the ‘external torque’ and the two-way ‘group × external’ torque interaction. We separately analyzed the trials 4–8 of each block to assess the extent of motor learning as well as of the respective first trials of blocks 2–4 to investigate the transfer of motor learning after a CoM change.

The fixed effects predictors of the models for the pseudorandom sequence condition were: the ‘external torque’, the ‘group’, the ‘CoM action’ (CoM-retained/inverted) and the resulting two- and three-way interactions ( ‘external torque × CoM action’, ‘external torque × group’, ‘group × CoM action’, ‘external torque × CoM action × group’).

We performed omnibus Wald-type F-tests of the model predictors with type-III analyses of variance (ANOVA) using the ‘lmerTest’ package^83^ as well as post-hoc t-Tests of pairwise comparisons between the hand-matched control and stroke groups (CL-SL, CR-SR) patient- and hand-matched control groups based on the marginal means of the LMMs with Holm-Bonferroni correction for multiple testing using the ‘emmeans’ package^84^. The predictor degrees of freedom of the LMMs were approximated with the Kenward-Roger method. It must be noted that the used hand used influences the ANOVA omnibus main effects of ‘group’ and the main interaction ‘external torque × group’. Therefore, statistical inferences on the impact of stroke on the torque planning were based on the results of the post-hoc pairwise comparisons controlling for the hand used. Initially planned analyses on the effect of apraxia and neglect on torque control could not be performed as too few patients showed signs of apraxia or neglect (see "Apraxia and neglect" section).

We performed a post-hoc power analyses for the torque variables in the no-cues-, blocked condition by calculating the power to detect group differences between 0.05 and 0.5 (steps of 0.05) with the alpha-level set to 0.25 using the ‘Superpower’ package in R^85^ (for details see Supplementary Figure S5).

### Ethics approval and consent to participate

The experimental procedures were approved by the Institutional Review Board of the School of Medicine at the Technical University of Munich and were in accordance with the Declaration of Helsinki. All subjects gave informed consent to participate in the study.

## Results

### Demographic characteristics, clinical measures and static coefficients of friction

We found no statistically significant differences between groups regarding age (*p* = 0.56), years since stroke onset (*p* = 0.51), gender distribution (*p* = 0.91), mRS (*p* = 0.70), mean coefficient of friction (*p* = 0.29, see also Supplementary Fig. S2), nor the voluntary maximum GF in the tripod grip (*p* = 0.609) (see Table 1).

### Apraxia and neglect

The vast majority of patients scored within the normal range in the administered apraxia and neglect tests: Only three patients with left MCA strokes scored below the cutoff in the hand imitation test (< 18), two of these patients (ID24, ID27) also failed the finger imitation (< 17)—and pantomime tests (< 45, Supplementary Fig. S1). Regarding the paper-based tests of hemispatial-neglect, only one patient with right MCA stroke (ID9) showed a line bisection deviation suggestive of hemispatial neglect to the left. However, results of the letter cancellation test were within the normal range in all patients. The results of the hand- (*p* = 0.070), and finger imitation tests (*p* = 0.616), the pantomime score (*p* = 0.241) as well as the line bisection (*p* = 0.43) and the CoC on the letter cancellation test (*p* = 0.201) did not differ between patient groups. The only significant difference between the SL and SR group was found for the percentual left–right reaction time difference in the Posner test (*p* = 0.001). Whereas SL patients were about 9.1% (SD 8.3%) slower in reacting to a stimulus on the right side, SR patients were 14.6% (SD 16.5%) slower when the stimulus was on the left side. In contrast, the mean reaction time in the Posner test (*p* = 0.35) was similar between patient groups. Table 1 summarizes the demographic, clinical and grip related measures of the participant groups.

### Torque compensation at lift off

#### No cues, blocked condition trials 4–8: Sensorimotor learning of the anticipatory coordination of centers of pressure and grip force is spatially biased following stroke

Participants of all groups only needed some 2–3 lift trials to learn to compensate for torques at the moment of lift-off. After that, Tcom remained stable for the rest of the block (see Supplementary Fig. S3 for the individual and group-averaged Tcom trajectories across trials in the ‘no cues’ condition).

**Figure 2:**
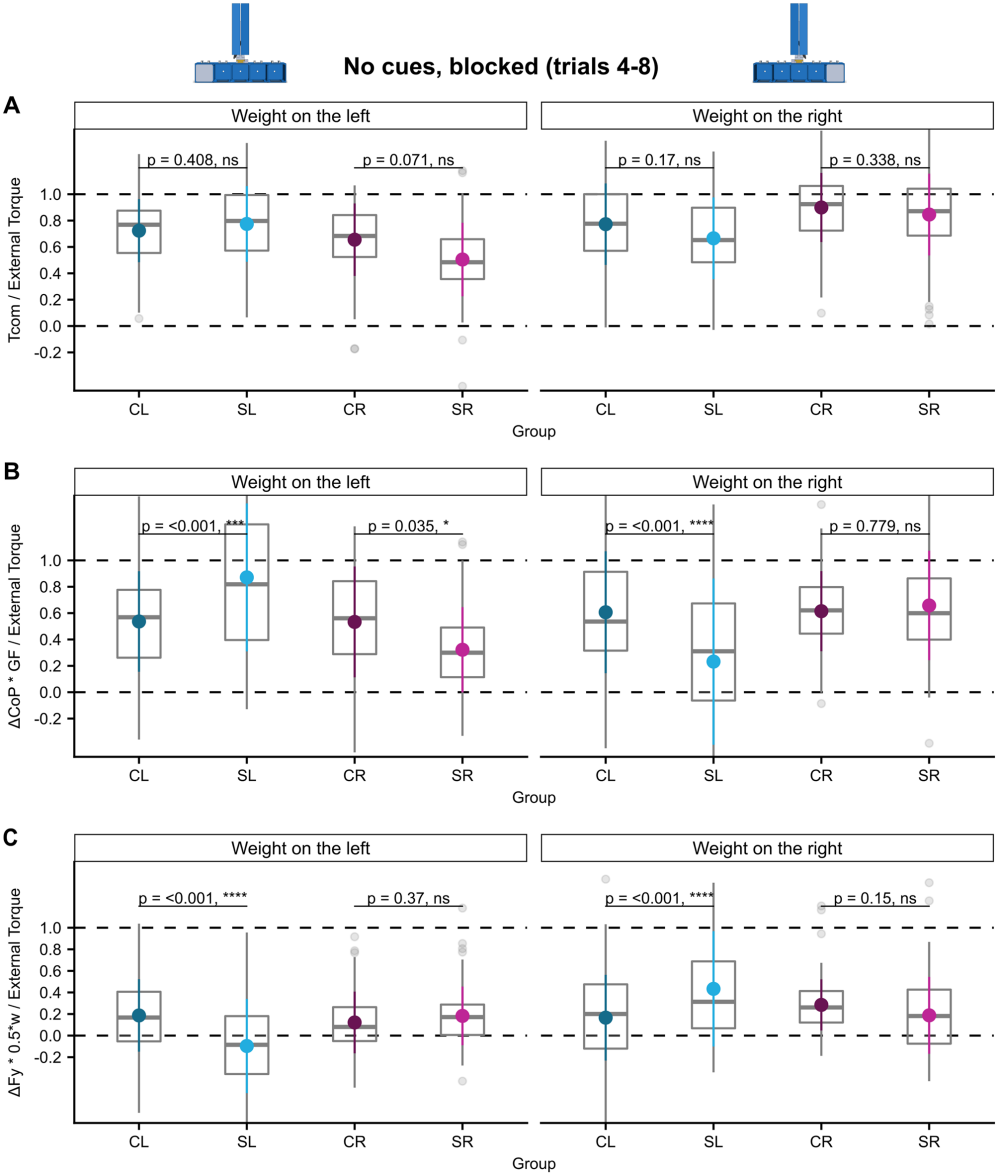
Sensorimotor learning of anticipatory torque compensation. Box and whiskers plots in the style of Tukey (central horizontal line: median, lower, and upper hinges: 25th and 75th percentiles, upper and lower whiskers extend up to 1.5 interquartile ranges) as well as the mean and standard deviation of the ratios of anticipatory torque anticipation success Tcom/external torque (**A**), ΔCoP * GF/External Torque (**B**), and ΔFy * 0.5*w/External Torque (**C**) for trials 4–8 of blocks in the ‘no cues’ condition are depicted for each group together with Holm-adjusted *p*-values of post-hoc t-tests of pairwise differences between controls and left- respectively right-hemispheric stroke patients.

All groups generated similar compensatory torques at lift-off in trials 4–8 with no significant differences between stroke and control groups (main effect of ‘group’ n.s., significant ‘ext. torque × group’ interaction’ F (3, 858)  = 33.2, *p* < 0.001, see Supplementary Table S2, no significant post-hoc comparison of interest). However, there was a trend towards a decreased Tcom for the SR-group when the weight was on the left side which was not significant after Holm-correction (t (55.3) =  − 2.15, *p* = 0.071, see Fig. 2A and Supplementary Table S3).

In contrast, the torque components at lift-off were spatially biased following a specific directional pattern in both stroke groups depending on the external torque (main effect ‘group’ n.s., significant ‘ext. torque × group’ interaction’ F (10.1, 858) = 53.7, *p* < 0.001, see Supplementary Table S4). The torque generated by grip force being produced at different vertical finger positions $\left( {\frac{{\Delta {\text{CoP }}*{\text{GF}}}}{{{\text{External}}\,{\text{Torque}}}}} \right)$ was lower in the SL group than the CL group when the CoM was on the right, i.e. contralesional, side (post-hoc comparison SL-CL: estimate =  − 0.37, ηp2 = 0.24, t (65.5) =  − 4.59, *p* < 0.001, see Fig. 2B and Supplementary Table S5) but higher than in the CL group when the weight was on the left, i.e. ipsilesional, side (post-hoc comparison SL-CL: estimate = 0.33, ηp2 = 0.20, t (65.5) = 4.10, *p* < 0.001). The torque produced by different load forces at the handle sides was biased in the opposite direction (main effect ‘group’ n.s., significant ‘ext. torque × group’ interaction’ F (3, 858) = 29.3, *p* < 0.001, see Supplementary Table S6), i.e. $\frac{{\Delta {\text{Fy}}*{\text{w}}/2}}{{{\text{External}}\,{\text{Torque}}}}$ was higher in the SL- than the CL group for a CoM on the contralesional, right side (post-hoc comparison SL-CL: estimate = 0.27, ηp2 = 0.20, t (98.2) = 5.03, *p* < 0.001, see Fig. 2C and Supplementary Table S7) and lower for a CoM on the ipsilesional, left side (post-hoc comparison SL-CL: estimate =  − 0.28, ηp2 = 0.22, t (98.2) =  − 5.33, *p* < 0.001). As the patterns of the object-centered spatial bias are diametrically opposed for $\frac{{\Delta {\text{CoP}}*{\text{GF}}}}{{{\text{External}}\,{\text{Torque}}}}$ and $\frac{{\Delta {\text{Fy}}*{\text{w}}/2}}{{{\text{External}}\,{\text{Torque}}}}$ the effects seem to cancel each other out resulting in normal total torques (Tcom) as outlined above.

SR patients equally exerted less torque by grip force being produced at different vertical finger positions $\left( {\frac{{{{\Delta {\text{CoP}} *{\text{GF}}}}}}{{\text{External Torque}}}} \right)$ than CR controls when the CoM was on the contralesional, left side (post-hoc comparison SR-CR: estimate =  − 0.22, ηp2 = 0.06, t (67.4) =  − 2.16, *p* = 0.035 see Fig. 2B and Supplementary Table 5), however $\frac{{\Delta {\text{CoP}}*{\text{GF}}}}{{{\text{External}}\,{\text{Torque}}}}$ was not increased for the ipsilesional CoM side and we found no differences of the torque produced by differential load forces $\left( {\frac{{\Delta {\text{Fy}}*{\text{w}}/2}}{{{\text{External}}\,{\text{Torque}}}}} \right)$ at lift off in the SR group.

#### No cues, blocked condition, trials after CoM change: Failed transfer of sensorimotor memories to explicit CoM changes.

**Figure 3:**
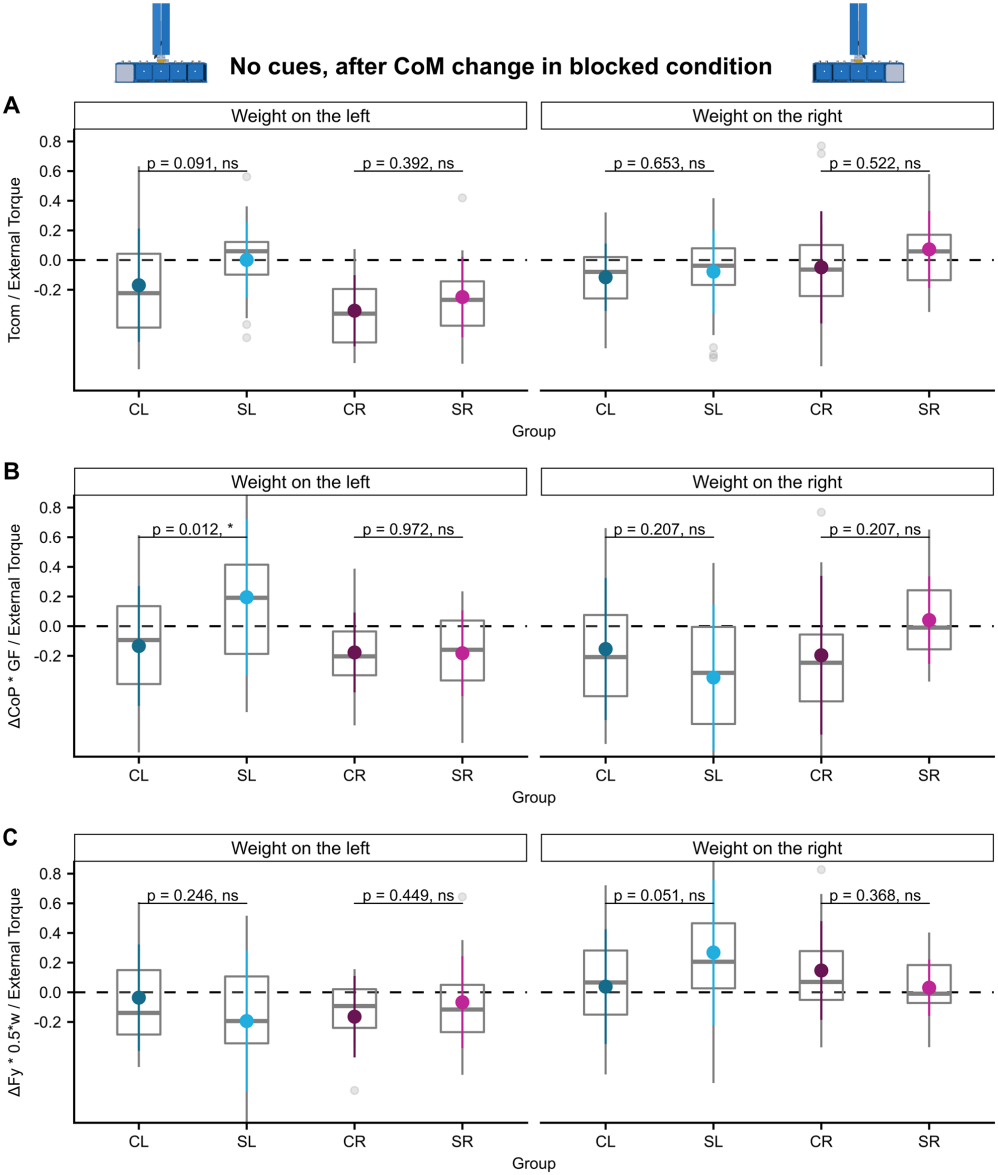
Transfer of sensorimotor learning of anticipatory torque compensation to explicit CoM changes. (**A**) Tcom/external torque, (**B**) ΔCoP * GF/External Torque, and (**C**) ΔFy * 0.5*w/External Torque of the first trial of a block after the CoM has changed in the ‘no cues, blocked’ condition (first trial of first block excluded) of all groups.

Despite being explicitly told that the CoM would be changed to the opposing side at the end of each block of eight trials, subjects of all groups subsequently failed to adapt to the new CoM situation and could not inverse the direction of the previously learned Tcom, i.e. transfer sensorimotor memories. This stands in line with previous studies [e.g.^52,78^]. Tcom was mostly near zero but clearly generated in the wrong, i.e. the previously learned, direction as indicated by a negative ratio of $\frac{{{\text{Tcom}}}}{{{\text{External }}\,{\text{Torque}}}}$. We observed no significant Tcom differences between stroke and control groups (main effect ‘group’ n.s., significant ‘ext. torque × group’ interaction’ F (3, 858)  = 33.2, *p* < 0.001, no significant post-hoc comparisons, see Fig. 3A and Supplementary Tables 8 and 9). Concerning the torque components (main effect ‘group’ n.s., significant ‘ext. torque × group’ interaction’ F ( 3, 858) = 53.7, *p* < 0.001, see Supplementary Table S10), the SL group applied a higher torque by grip force being exerted at different vertical finger positions $\left( {\frac{{\Delta {\text{CoP}}*{\text{GF}}}}{{{\text{External}}\,{\text{Torque}}}}} \right)$ than controls when the hidden weight was transferred to the ipsilesional, left side (post-hoc comparison SL-CL: estimate = 0.33, ηp2 = 0.06, t (126.2) = 2.81, *p* = 0.012, see Fig. 3B and Supplementary Table S11). Apart from this, there were no further differences between stroke- and control groups (see also Fig. 3C and Supplementary Table S13).

#### No cues, pseudorandom condition: Torque planning according to sensorimotor memories despite uncertainty

**Figure 4:**
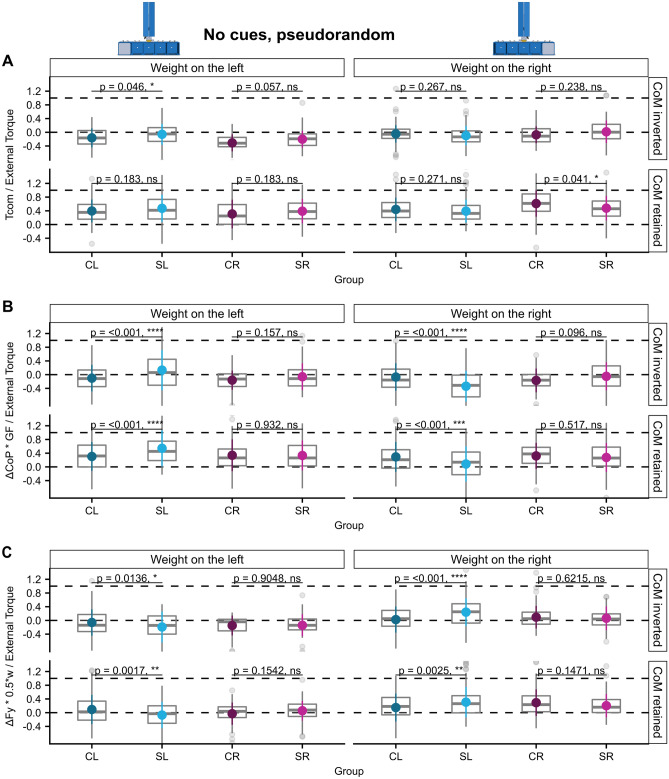
Sensorimotor torque control in uncertainty. (**A**) Tcom/external torque, (**B**) ΔCoP * GF/External Torque, and (**C**) ΔFy * 0.5*w/External Torque of all groups averaged for trials in which the CoM has changed and trials in which it remained constant for both possible CoMs in the ‘no cues, pseudorandom’ condition.

In this condition the position of the hidden weight was either retained or inverted between trials in a pseudorandom fashion. After each trial the hidden weight was removed and placed back either into the same or the opposite position. Although a rational torque planning was not possible in this condition, we observed that all groups planned according to the previous lifts resulting in clearly positive Tcom ratios when the CoM was not inverted (main effect ‘CoM action’: F (3, 1569)  = 1151.0, *p* < 0.001, see Supplementary Table S14 and Fig. 4A). Remarkably, we did not observe the generation of Tcom of similar magnitudes directed in the wrong direction following a CoM inverse. Rather, Tcom was close to zero in trials after a CoM inversion suggesting that participants must have partially corrected the exerted torque already until lift-off. We found two just significant post-hoc group differences reflecting significant ‘ext. torque × group’ (F (3, 1569)  = 18.1, *p* < 0.001) and ‘CoM-action × group’ [F (3, 1569)  = 3.8, *p* = 0.01] interactions. First, the SL group exerted a Tcom closer to zero when the CoM was switched to the left (post-hoc comparison SL-CL: estimate = 0.1, ηp2 = 0.01, t (236.9) = 2.28, *p* = 0.046, see Fig. 4A and Supplementary Table S15). Secondly, the SR group produced a smaller Tcom when the CoM remained on the right (post-hoc comparison SL-CL: estimate =  − 0.13, ηp2 = 0.02, t (246.7) = 2.33, *p* = 0.041).

Concerning the torque components, we again found contrasting object-centered spatial biases of the torque anticipation strategies in the SL group when compared with the CL group and this was irrespective of whether the weight position was changed or not (main effect ‘group’ n.s., significant ‘external torque × group’ interaction’ F (3, 1569)  = 30.2, *p* < 0.001, interaction ‘CoM-action × group’ n.s., see Supplementary Table S16): While the torque generated by grip force being exerted at different vertical positions $\left( {\frac{{\Delta {\text{CoP}}*{\text{GF}}}}{{{\text{External }}\,{\text{Torque}}}}} \right)$ was less adequate (smaller ratio) when the weight was on the right, i.e. contralesional, side (post-hoc comparison SL-CL, CoM inverted: estimate =  − 0.27, ηp2 = 0.03, t (536.5) =  − 4.79, *p* < 0.001, CoM retained: estimate =  − 0.21, ηp2 = 0.03, t (531)  =  − 3.72, *p* < 0.001, see Fig. 4B and Supplementary Table S17) but more adequate (higher ratio) than in the CL group when the weight was on the left, i.e. ipsilesional side (post-hoc comparison SL-CL, CoM inverted: estimate = 0.23, ηp2 = 0.03, t (529) = 4.22, *p* < 0.001, CoM retained: estimate = 0.24, ηp2 = 0.03,t (529) = 4.28, *p* < 0.001). Again, the torque generated by differential load forces between sides was biased in the opposite direction (main effect ‘group’ n.s., significant ‘external torque × group’ interaction’ F (3, 1569)  = 16.9, *p* < 0.001, interaction ‘CoM-action × group’ n.s., see Supplementary Table S18), i.e. $\frac{{\Delta {\text{Fy}}*{\text{w}}/2}}{{{\text{External}}\,{\text{Torque}}}}$ was higher in the SL than in the CL group for a CoM on the contralesional, right side (post-hoc comparison SL-CL, CoM inverted: estimate = 0.22, ηp2 = 0.02, t (537) = 4.47, *p* < 0.001, CoM retained: estimate = 0.16, ηp2 = 0.02, t (531) = 3.25, *p* = 0.0025, see Fig. 4C and Supplementary Table S19) and lower for a CoM on the ipsilesional, left side (post-hoc comparison SL-CL, CoM inverted: estimate =  − 0.13, ηp2 = 0.02, t (529) =  − 2.72, *p* = 0.014, CoM retained: estimate =  − 0.16, ηp2 = 0.02, t (529) = 3.35, *p* = 0.002). No significant differences were detected between the right-hand groups SR and CR.

#### Geometric cues: successful torque anticipation in all experimental conditions

**Figure 5:**
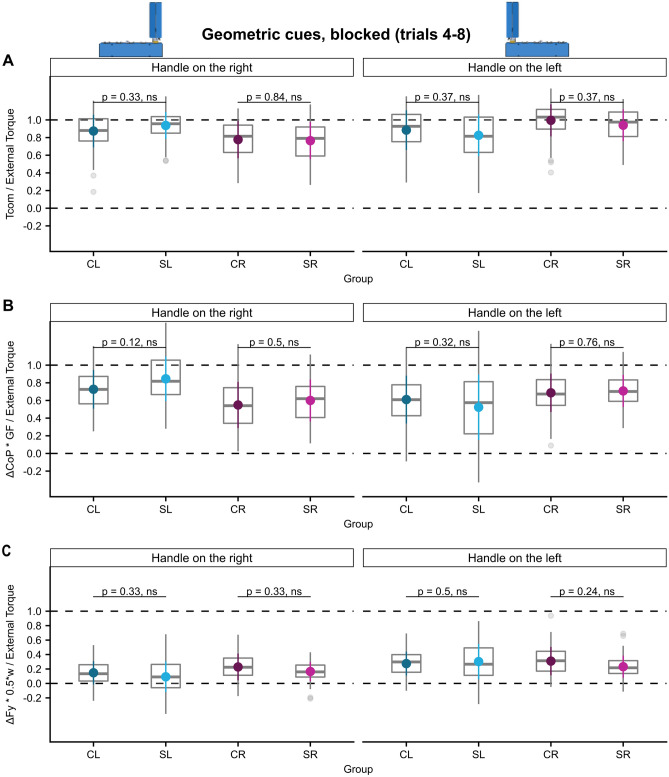
Learning of anticipatory torque compensation according to both geometric cues and sensorimotor memories. (**A**) Tcom/external torque, (**B**) ΔCoP * GF/External Torque, and (**C**) ΔFy * 0.5*w/External Torque for trials 4–8 of blocks in the ‘geometric cues’ condition of all groups.

**Figure 6:**
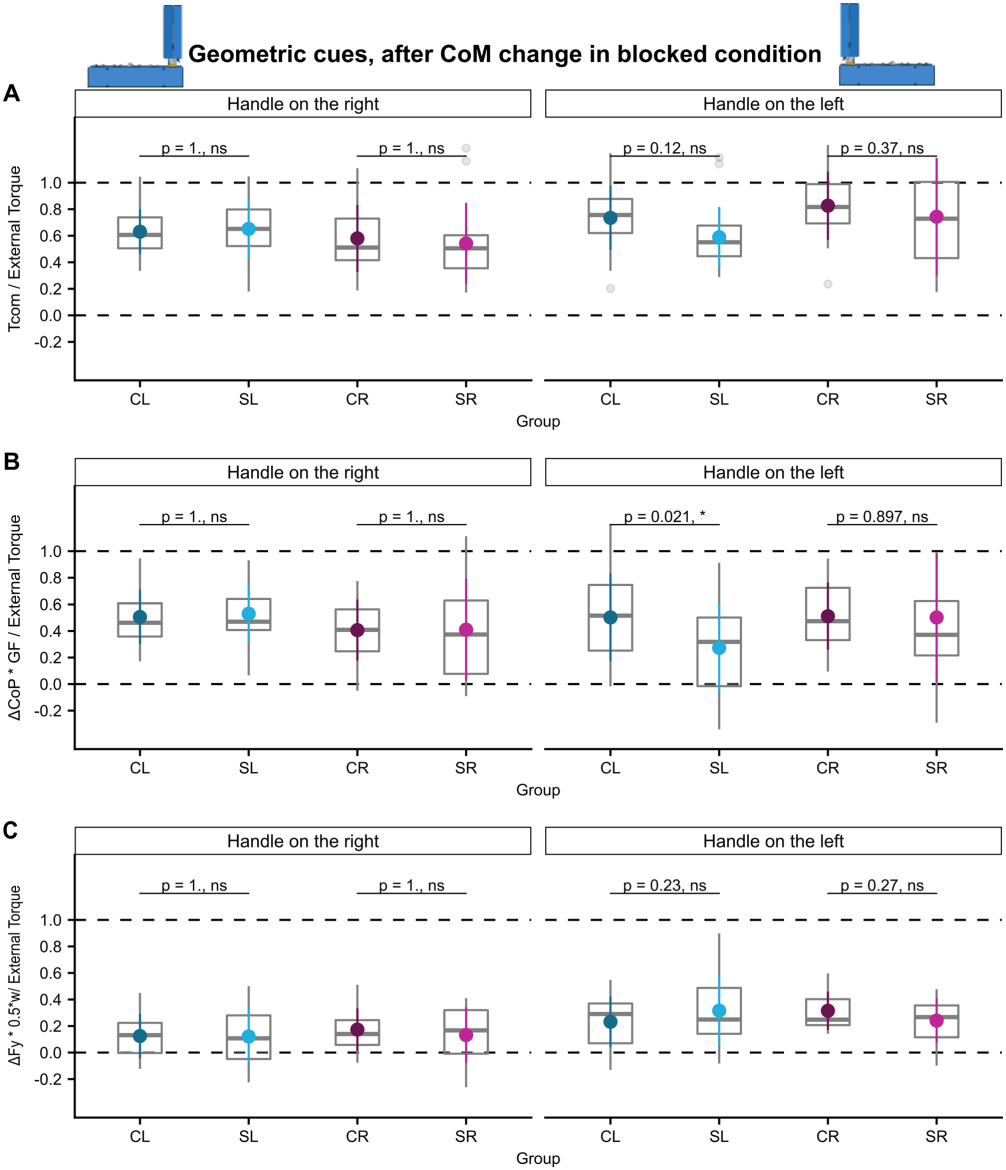
Interaction of visuomotor transformations and the transfer of sensorimotor learning of anticipatory torque compensation after CoM change in the blocked condition. (**A**) Tcom/external torque, (**B**) ΔCoP * GF/External Torque, and (**C**) ΔFy * 0.5*w/External Torque of the first trial of a block after the CoM has changed in the ‘geometric cues’ condition (first trial of first block excluded) of all groups.

**Figure 7:**
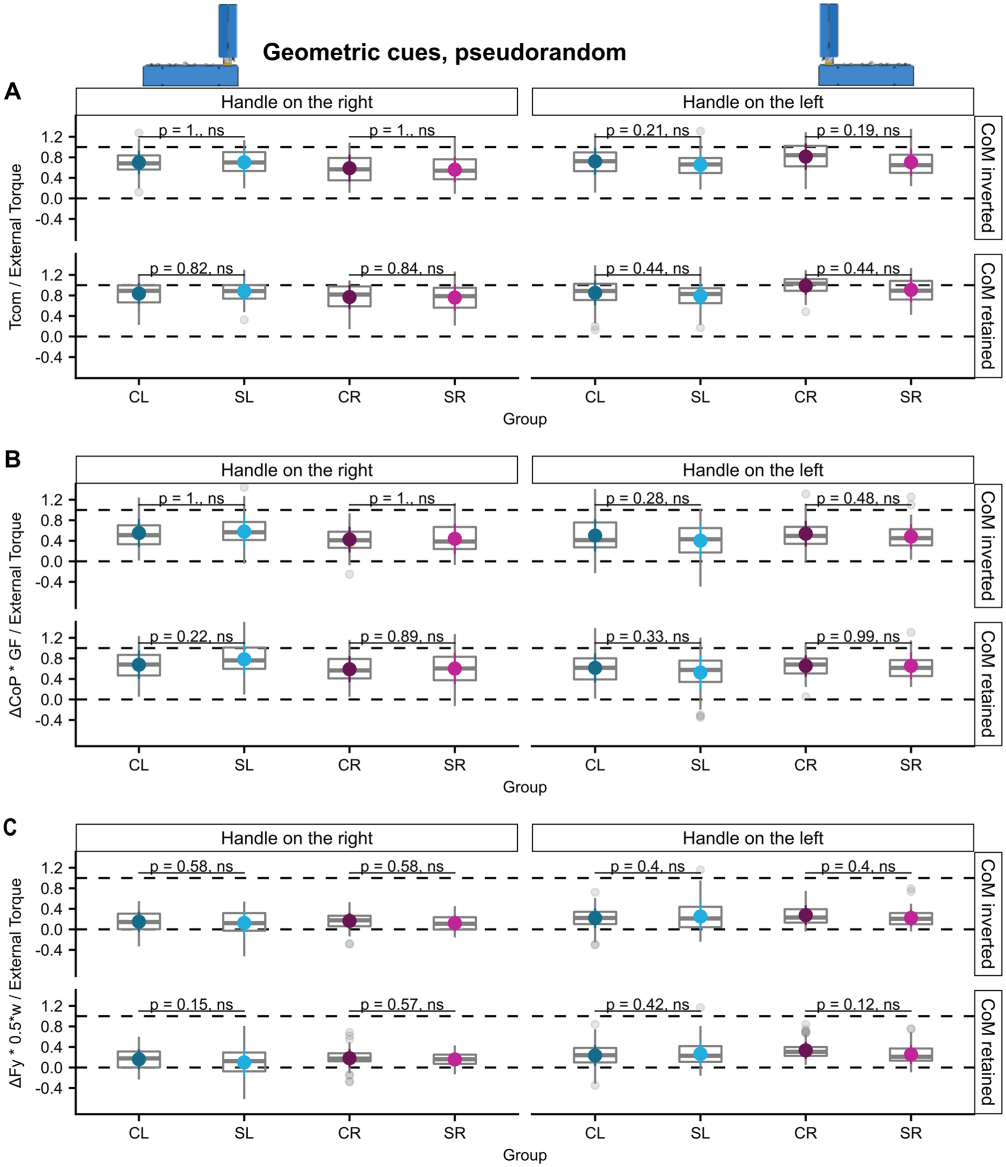
Interaction of visuomotor transformations and the transfer of sensorimotor learning of anticipatory torque compensation after CoM change in the pseudorandom condition. (**A**) Tcom/external torque, (**B**) ΔCoP * GF/External Torque, and (**C**) ΔFy * 0.5*w/External Torque of all groups averaged for trials in which the CoM has changed and trials in which it remained constant for both possible CoMs in the ‘geometric cues, pseudorandom’ condition.

In the geometric cue condition in which the CoM was altered by attaching the handle either on the left or right edge of the base participants of all groups successfully compensated for the arising external torque at lift off both in the blocked as well as in the pseudorandom condition and even in trials following a change of the handle position in the blocked condition. Supplementary Fig. S4 depicts the Tcom trajectories of all participants in the geometric-cue conditions. Tcom was mostly generated by GF being produced at different vertical centers of pressure and only to a lesser degree by differential load force sharing. We found no differences of Tcom success in post-hoc comparisons between the stroke and control groups in neither the blocked- nor the pseudorandom condition despite significant ‘ext. torque × group’ interactions (see Figs. 5, 6, 7 and Supplementary Tables S20–36). This lack of group differences was also observed when analyzing the torque components, with the exception of the finding of less successful torque generation by $\frac{{\Delta {\text{CoP}}*{\text{GF}}}}{{{\text{External}}\,{\text{Torque}}}}$ in the SL group than the CL group in the first trials in the blocked condition following a change of the handle to the left, i.e. the CoM to the right side (post-hoc comparison SL-CL: estimate =  − 0.23, ηp2 = 0.07, t (96)  =  − 2.60, *p* = 0.021, see Fig. 6B and Supplementary Table S29).

#### ∆CoP and GF at lift-off

**Figure 8:**
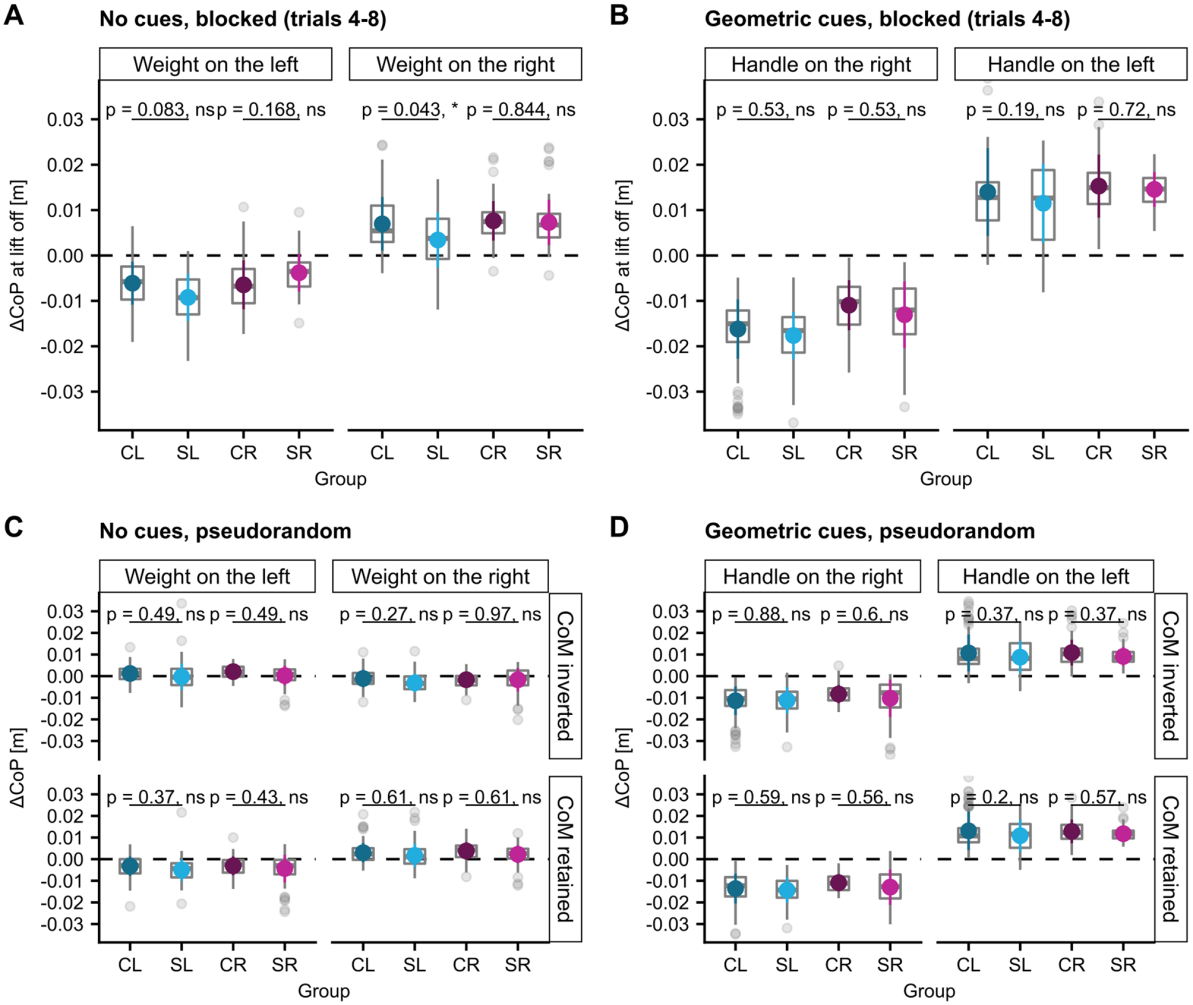
∆CoP at lift-off. (**A**) the blocked, no-cues condition (trials 4–8), (**B**) the blocked, visual-cues condition (trials 4–8), (**C**) the pseudorandom, no-cues condition and (**D**) the pseudorandom, visual-cues condition.

**Figure 9:**
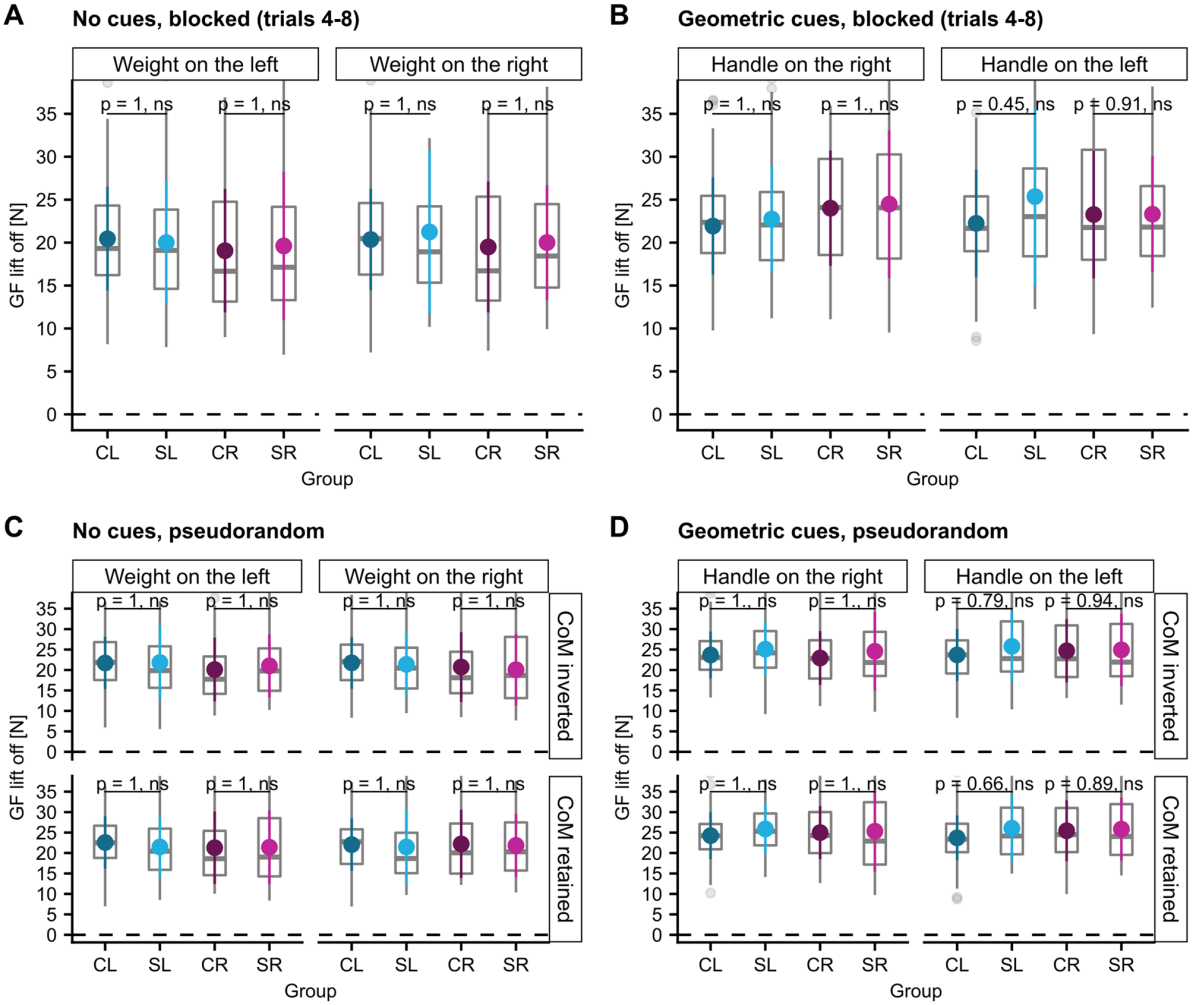
GF at lift-off was similar between stroke and control groups in all experimental conditions. (**A**) the blocked, no-cues condition (trials 4–8), (**B**) the blocked, visual-cues condition (trials 4–8), (**C**) the pseudorandom, no-cues condition and (**D**) the pseudorandom, visual-cues condition.

The total compensatory torque and its components at lift-off were the task-level variables participants had to control to prevent object tilt. While the load force sharing between grasp-sides, ∆Fy, is directly proportional to the resulting torque component as the grip width is constant, both the center of pressures and the GF must be actively controlled to achieve the desired torque product ∆CoP*GF. Therefore, we were interested to evaluate whether the found spatial biases of the torque produced by vertical center of pressure modulation, ∆CoP*GF, can be traced back to distinct alterations in the control of either ∆CoP, GF or both at lift-off. Regarding ∆CoP, we found a non-significant trend toward a better modulation in the SL than the CL group when the weight CoM was on the left side (post-hoc comparison SL-CL: t (46.0) =  − 2.1, *p* = 0.083, see Supplementary Tables 39) and a significantly worse modulation when the CoM was on the right side (post-hoc comparison SL-CL: estimate =  − 0.004 m, ηp2 = 0.11, t (46)  =  − 2.38, *p* = 0.043, see Fig. 8A). These findings are consistent with the reported results for ∆CoP*GF, although less robust. Apart from that, there were no other significant differences between groups in post-hoc testing (see Fig. 8 and Supplementary Tables 37–44). Concerning GF, we did not detect any significant differences between stroke and control groups in post-hoc testing (see Fig. 9 and Supplementary Tables 45–52).

#### Post-hoc power analysis

We decided on our sample size pragmatically. The final sample size was determined by the maximum number of patients with stroke we could feasibly recruit and test given financial and time constraints. We performed a post-hoc sensitivity power analysis for the main outcome measures $\frac{{{\text{Tcom}}}}{{\text{External Torque}}}$,$\frac{{{{\Delta {\text{Fy}}* {\text{w}}}}/2}}{{\text{External Torque}}}$ and $\frac{{{{\Delta {\text{CoP}} *{\text{GF}}}}}}{{\text{External Torque}}}$ in the no cues, blocked condition. The outcome variables were repeatedly centered for each group (separately for both external torques) to yield group differences between the stroke and control groups between 0.05 and 0.5 in steps of 0.05. The final sample size meant that the study was able to reliably detect a post-hoc estimated marginal means group difference in $\frac{{{\text{Tcom}}}}{{{\text{External }}\,{\text{Torque}}}}$ and $\frac{{\Delta {\text{Fy}}*{\text{w}}/2}}{{{\text{External }}\,{\text{Torque}}}}$ of 0.2 and a difference of 0.3 in $\frac{{\Delta {\text{CoP}}*{\text{GF}}}}{{{\text{External }}\,{\text{Torque}}}}$ between the ‘CL’ and ‘SL’ groups as well as differences of 0.25 $\left( {\frac{{{\text{Tcom}}}}{{{\text{External}}\,{\text{Torque}}}}{\text{and}}\,\,\frac{{\Delta {\text{Fy}}*{\text{w}}/2}}{{{\text{External }}\,{\text{Torque}}}}} \right)$ and 0.35 $\left( {\frac{{\Delta {\text{CoP}}*{\text{GF}}}}{{{\text{External }}\,{\text{Torque}}}}} \right)$ between the ‘CR’ and ‘SR’ groups with an alpha of 0.025, and > 80% power (see Supplementary Fig. S5 for details).

## Discussion

This study was set out to investigate whether manual torque control with the ipsilesional hand is impaired in patients in the chronic stage following unilateral stroke when lifting objects. Using a cross-over design with two cue- and two-sequence conditions, we studied both a cue-condition in which learning had to rely on previous sensorimotor memories of recent lifts as well as a visual cue condition in which the object CoM could be inferred from object geometry. Moreover, participants performed trials both in blocked, i.e. predictable, sequence-condition as well as a pseudorandom sequence condition in which the CoM could change after each trial in an unforeseeable manner. Both our main hypotheses that (a) ∆CoP modulation was impaired in the SR group and (b) deficient load force sharing (∆Fy) in the SL group would lead to impaired torque compensation at lift-off, were not confirmed. Instead, both stroke groups learned to compensate torques at lift-off to overall similar degrees as controls in both cue conditions and patients presented neither general deficits of force-to finger position coordination, nor elevated GF levels, on a group level.

Instead, we observed a specific pattern of an object-centered spatial bias of torque components in patients with stroke when having to rely on sensorimotor memories. While torques resulting from force being produced at different vertical finger positions, ∆CoP × GF, were lower when the object CoM was on the contralesional side and higher when the CoM was on the ipsilesional side in patients with left hemispheric stroke, torques generated by differential load forces between sides (∆Fy × w/2) were biased in the opposite direction. These biases largely cancelled each other out. SR patients also applied a distinctly smaller ∆CoP × GF for a CoM on the contralesional, left side but showed neither a clear compensation by ∆Fy × w/2 nor an increase in ∆CoP × GF for a CoM on the ipsilesional side. Torque control was intact in both stroke groups when a geometric cue on the weight distribution was available.

We summarize and discuss our findings in the following sections.

### Preserved sensorimotor force-to position coordination despite a spatial bias of ∆CoP × GF following stroke

In line with studies of young and elderly healthy adults^52,62,78^, participants in all groups quickly learned to exert an adequate Tcom when the CoM was constant across the trials of a block. At the beginning of a new block they failed to transfer the learned torque planning to the new situation even when they were explicitly told that the CoM would be inverted. They also continued to rely on sensorimotor memories of previous lifts when the CoM could change from trial to trial^53,54^. Intriguingly, the magnitude of torques directed in the wrong direction when the CoM had unexpectedly changed from one side to the other was smaller than the torque exerted in the right direction when the CoM had stayed the same. This suggests that participants in all groups applied corrective feedback-mechanism to partially correct for erroneous torque anticipation within the short time interval prior to lift-off, although full feedback about object torque only becomes available after lift-off. This finding is consistent with our previous studies in healthy subjects^53,57^ and the time course and underlying mechanism of these corrections need to be further explored in future analyses. There were no noteworthy differences of Tcom between the stroke and the control groups, despite the emergence of a distinct pattern of differences between the torque components.

The most remarkable finding of this study is that the torque resulting from grip force being produced at different vertical centers of pressure, ∆CoP × GF, and from differential load force sharing between sides, ∆Fy × w/2, were spatially biased in diametrical directions in patients with left hemispheric-stroke when participants had to exclusively rely on sensorimotor memories to guide torque control: Patients with left hemispheric stroke applied a smaller ∆CoP × GF at lift off than controls when the CoM was on the contralesional side but a higher ∆CoP × GF when the CoM was on the ipsilesional, i.e. left, side. In contrast, the torque resulting from differential load forces at the handle sides (∆Fy × w/2) was spatially biased in the opposite direction in SL-patients, i.e. ∆Fy × w/2 was higher for a CoM on the right- and lower for a CoM on the left side. As a consequence, the overall Tcom did not significantly differ between left hemispheric stroke patients and controls on the group level.

Patients with right hemispheric stroke also exhibited a markedly smaller torque resulting from grip force being produced at different vertical finger positions, ∆CoP × GF, but showed no signs of a compensatory load force distribution (∆Fy × w/2). However, this only translated to a not significant trend towards a lower Tcom. This was not significant after Holm correction as the variability was high and the sample size low. No significant differences or even visually discernible trends were found for Tcom or the torque components when the covert weight was on the ipsilesional right side.

As the center of pressure in the employed three-finger precision grip mostly depended upon the finger positioning when grasping the handle and to a lesser degree on the normal force distribution between the index and middle finger^79^, the torque component ∆CoP × GF arguably better represents explicit context-dependent motor planning in unconstrained grasping; whereas the load force distribution contributing to the total torque (∆Fy × w/2) is modulated as a function of finger-positioning after the formation of the grasp to achieve a targeted total torque^50,58,86–88^. Consequently, the observed spatial bias of load force sharing in left hemispheric stroke patients might represent a compensatory mechanism to counteract the spatial bias of grip force exerted at different vertical positions. This supports the concept of a task-level, i.e. high-level, neural representation of the task goal, namely the compensatory total torque, which is used to orchestrate both the feedforward as well as feedback control of the positions and forces of the low-level effectors, e.g. fingertips^49,54,89^.

However, the same pattern of spatial bias was evident in patients with left hemispheric stroke in the pseudorandom, no cues-condition with more successful ∆CoP × GF for a CoM on the left and a less successful ∆CoP × GF for a CoM on the right as well as opposing findings for the torque component ∆Fy × w/2, both for trials in which the CoM was inverted and trials in which the CoM was retained. This might suggest that the object-centered spatial torque bias depended upon the current side of the CoM but not the CoM of the previous trial on which sensorimotor memories for torque planning are based on. This could cast doubt on whether the torque component ∆CoP × GF can really be regarded as measure of exclusively anticipatory planning. Instead, it might also be possible that the bias observed in the pseudorandom condition affected the corrections of the torque components ∆CoP × GF and ∆Fy × w/2 just prior to lift-off according to sensory feedback. However, the results of the models fit to analyze the pseudorandom condition were complex, the standard errors high and the standardized effect sizes of significant group comparisons low. Consequently, one must be cautious in interpreting theses significant findings. In any case, it might be advisable to speak of a bias of torque control instead of torque anticipation, which implies exclusive feedforward control.

Irrespective of the relative contribution of feedforward- and feedback-mechanisms on torque generation at lift off, the opposingly directed object-centered spatial bias for ∆CoP × GF and ∆Fy × w/2 in left hemispheric stroke patients and the isolated bias for ∆CoP × GF in right hemispheric stroke patients corroborates the notion that different neural networks control these task level variables. This notion has previously been based on behavioral studies which could show that finger positioning represents context dependent, explicit, learning, whereas load force distribution is more influenced by effector- and use-dependent, implicit, learning processes^52,90^.

### Visuomotor processing of geometric cues for torque control is intact in chronic stroke patients

When the mass distribution could be inferred from the geometric shape of the object (L-Shape) all participant groups successfully compensated for torques arising at lift off mainly by adequately modulating the centers of pressure on both grip sides (∆CoP × GF) both when learning successful manipulation over a course trials with constant object properties but also when object geometry and weight distribution changed randomly. Given a geometric cue, torques by load force partitioning (∆Fy × w/2) only contributed a small part of the total Tcom. Changing the object geometry after a sequence of 8 trials led to an interference of sensorimotor memories of previous lifts on lift planning resulting in a slightly smaller Tcom. The found successful processing of geometric cues to guide torques and the sensorimotor inference on geometric processing confirm previous studies examining young- and elderly healthy subjects^56,57,79^.

The compensatory torque and torque components did not differ in the stroke groups suggesting intact visuomotor processing of object shape to infer mass distribution. This stands in line with previous studies which showed that grip force scaling according to object size was not affected by unilateral MCI stroke on a group level^63,64,91^. Most notably, we found no evidence of a spatial bias of the torque components ∆CoP × GF or ∆Fy × w/2 in the stroke groups suggesting that these biases following stroke are specific to sensorimotor control and can be corrected by visual control.

### Evidence for an allocentric premotor neglect?

The finding of an object-centric spatial bias of the sensorimotor torque control with a higher than normal ∆CoP × GF for a CoM on the ipsilesional side (only SL group) and a lower ∆CoP × GF for a CoM on the contralesional side (both stroke groups) could be taken as evidence for a shift of spatial attention towards eccentric loads on the ipsilesional side and away from loads on the contralesional side following unilateral stroke. This may represent a novel subtype of allocentric premotor attention bias, i.e. neglect. Concerning the association between neglect and motor control, the phenomenon of premotor neglect (PMN), i.e. an intentional, voluntary, and directional motor disorder of movements in or to the contralesional space which equally affects the limbs on both sides following stroke^92^. Patients show an abnormal movement initiation (hypo- or akinesia) as well as slowed (bradykinesia) and hypometric reaching movements towards goals in their contralesional hemispace even when tested with their ipsilesional hand^93–96^. Moreover, they deviate towards the ipsilateral side when pointing straight ahead when blindfolded which is suggestive of a shift in the egocentric reference frame^97,98^. It is important to note, however, that participants in our study were allowed to adjust the exact position and orientation of the object on the table in a way that allowed for comfortable grasping. Usually, the object was positioned in the hemispace of the involved, ipsilesional hand. Therefore, in contrast to previous studies on premotor neglect the reference frame of torque control in the current study was rather object- or hand specific, i.e. allocentric, than egocentric. To the best of our knowledge, a signs of premotor-allocentric neglect have not yet been reported for an everyday object manipulation task.

As the found bias concerns the control of object tilts due to a directed allocentric eccentric load, studies investigating the perception of the subjective vertical and -horizontal might also be relevant to the interpretation of our findings. These studies revealed that patients with left-sided as well as right-sided neglect systematically tilted the spatial orientation of the subjective vertical- and horizontal in the direction of the neglected, contraversive, side both in a visual and tactile modality- suggesting multisensory spatial orientation deficits in neglect patients^99–102^. Applied to our studied task, a shift of the targeted subjective vertical of the object handle towards the contralesional side might have led to the tendency of an under compensation of torques towards the contraversive side and to an over compensation of torques towards the ipsiversive side, as a small tilt to the contralesional side might have been perceived as ideal. However, we found this only to be true for the anticipatory torque component ∆CoP × GF, but not for the torque resulting from asymmetric load force sharing (∆Fy × w/2). Moreover, we only found evidence for a bidirectional spatial bias in patients with left hemispheric stroke while patients with right hemispheric stroke only showed a decreased ∆CoP × GF for a CoM on the contralesional side but no ∆CoP × GF elevation when the CoM was on the ipsilesional side.

None of the chronic stroke patients exhibited clear signs of perceptual hemispatial neglect in the conducted pen-and-paper based tests. As we did not expect to find an object centered bias of torque control we unfortunately did not test for the presence of an allocentric neglect. Nevertheless, our finding could be viewed a subtle form of an object centered premotor attention bias regarding torques. However, this inattention might not be of relevance in daily living in the majority of stroke patients as both intact load-force coordination and visuomotor processing of object geometry can compensate for the bias.

### Future research directions

Future clinical-experimental studies should aim to further investigate the association between perceptual and motor manifestations of neglect and torque control in object manipulation following stroke. The motor manifestations of neglect comprise both premotor- and motor neglect, the latter being defined as an underuse of the contralesional side of the body in the absence of—or out of proportion to—weakness or sensory impairments^92,103,104^. To this end, larger cohorts of stroke patients with unilateral cortical lesions seen on MRI-imaging in the acute stage of stroke should be included as the prevalence of motor neglect is estimated to range between 12 and 33% of patients with acute stroke and some 8% of patients with chronic stroke^105,106^. The prevalence of premotor neglect remains unclear as clinical tests of premotor neglect [e.g. Milner- or Bisiach- landmark tests^107^] might not be reliable^108^. Patients should be assessed for sensorimotor impairments, both egocentric- and allocentric visual neglect, personal neglect, the subjective vertical as motor- and premotor neglect. An ideal protocol to improve the understanding of torque control impairments in object manipulation following stroke should use a crossed-design investigating both hands (influence of sensorimotor impairments and/or motor neglect), object positions in both hemispaces (egocentric premotor neglect), object weight distributions on both sides (allocentric premotor neglect) as well as both a sensorimotor and geometric-visual cue condition (2 × 2 × 2 × 2 design). Voxel-based lesions symptom mapping analyses will help to uncover the neural correlates of the studied aspects of torque control.

### Study limitations

Finally, a number of limitations of this study must be considered. The main limitation is that the studied stroke groups were small and heterogenous regarding stroke type, localization, the time from stroke onset and the stroke related functional impairments. As only chronic stroke patients referred by outpatient therapists participated in this study we could only obtain the medical reports but failed to collect the CT or MRI imaging studies. Therefore, we cannot make claims on the role of specific neuroanatomical regions or networks in the studied tasks. Since our study is confined to highly chronic stroke patients, we cannot exclude that the pattern of torque control deficits differs in earlier phases of stroke. Moreover, we did not perform a comprehensive neurological exam. Since only few of the chronic stroke patients of the sample revealed clear signs of apraxia or neglect we could not analyze the impact of these syndromes on torque control. As we did not expect to find the object centered spatial bias of torque control a priori, we did not perform tests of allocentric neglect. Finally, it must be noted that we conducted numerous statistical tests of the primary and secondary variables of interest and experimental conditions rendering the analyses exploratory.

The current study is a pilot study which received no targeted funding and was conducted without a clinical partner. Therefore, the tested sample of patients with stroke was small and heterogenous. A post-hoc power analysis revealed that although the study seems to be appropriately powered to detect large group differences with sufficient power (in the no cues, blocked condition), it must be assumed that the study is underpowered to detect small and moderate effects.

However, despite the small samples size, patient heterogeneity and an exploratory statistical analysis plan a clear pattern of highly significant results emerged which reveal a novel aspect of impaired motor control of the ipsilesional hand following stroke and will guide the design of future studies on object manipulation following stroke.

## Conclusions

In summary, we found that patients with left-hemispheric stroke show a spatial bias of the torque resulting from grip force being applied at different vertical finger position depending on the object mass distribution when relying on sensorimotor memories with the torque component being increased for a CoM on the ipsilesional but decreased for a CoM on the contralesional side. This bias was compensated for by a load-force sharing biased in the opposite direction as evidence of intact force-to-position coordination. While patients with right hemispheric stroke also exhibited lower torques due to grip force being applied at different vertical finger position for a CoM on the contralesional side, we found no evidence for an increase of this torque component for a CoM on the ipsilesional side or a compensatory bias of load force distributions. When salient, congruent geometric cues were present, patient performance was not different from controls, suggesting that visuomotor processing ameliorates the noted sensorimotor bias. The sensorimotor object-centered spatial bias of torque strategies could be a subtle sign of a premotor attention bias, respectively a premotor attention bias as a subtype of neglect, which might be even present in the absence of a an evident hemispatial neglect. The found object centered spatial bias of torque controls should be further investigated in larger and more homogenous cohorts of stroke patients in the acute stage with a refined protocol designed to evaluate the association between premotor- and perceptual (allo- and egocentric) neglect and torque control.

## Supplementary Information

Supplementary Information.

## Data Availability

The data that support the findings of this study are openly available in “figshare” at 10.6084/m9.figshare.17057675 .
