# Supplementary material for: Object-centered sensorimotor bias of torque control in the chronic stage following stroke
Source: Sci Rep. 2022 Aug 25;12:14539. doi: 10.1038/s41598-022-18754-z (PMC9411611; doi:10.1038/s41598-022-18754-z)
Supplement: Supplementary file 1 — Supplementary Information. [file 41598_2022_18754_MOESM1_ESM.pdf]

Supplementary Material

Object-centered bias of sensorimotor torque control in the chronic stage following stroke

Thomas Rudolf Schneider[1,2]* and Joachim Hermsdörfer[1]

[1] Chair of Human Movement Science, Department of Sport and Health Sciences, Technical University of Munich, Georg-Brauchle-Ring 60/ 62 D-80992 Munich, Germany

[2] Department of Neurology, Cantonal Hospital of St. Gallen, Rorschacher Str. 95 CH - 9007 St. Gallen, Switzerland

1 List of Figures

Supplementary Figure S 1: Demographics and clinical test results in left- and right hemisphere stroke patients. 6

Supplementary Figure S 2: Coefficients of static friction as assessed by object slip trials. 7

Supplementary Figure S 3: Individual and group averaged trajectories of Tcom of all trials in the no cues condition depicted for each group. 8

Supplementary Figure S 4: Individual and group averaged trajectories of Tcom of all trials in the geometric cues condition depicted for each group. 9

Supplementary Figure S 5: Results of a post-hoc sensitivity power analysis for the main outcome measures in the no cues, blocked condition. 10

2 List of Tables

Supplementary Table S 1: Demographic and clinical information for all participating stroke patients. 11

Supplementary Table S 2: Type III analysis of variance table with Kenward-Roger's method of the linear mixed effects model of Tcom/ External Torque of trials 4-8 in the no-cues, blocked condition..13

Supplementary Table S 3: Post-hoc t-tests of pairwise comparisons between stroke patient- and hand-matched control groups based on the marginal means of the LMM of Tcom/ External Torque of trials 4-8 in the no-cues, blocked condition with Holm-Bonferroni correction for multiple testing applied 13

Supplementary Table S 4: Type III analysis of variance table with Kenward-Roger's method of the linear mixed effects model of ΔCoP x GF/ External Torque of trials 4-8 in the no-cues, blocked condition..13

Supplementary Table S 5: Post-hoc t-tests of pairwise comparisons based on the LMM of ΔCoP x GF/ External Torque of trials 4-8 in the no-cues, blocked condition. 13

Supplementary Table S 6: Type III analysis of variance table with Kenward-Roger's method of the linear mixed effects model of ΔFy x 0.5w/ External Torque of trials 4-8 in the no-cues, blocked condition..13

Supplementary Table S 7: Post-hoc t-tests of pairwise comparisons based on the LMM of ΔFy x 0.5w/ External Torque of trials 4-8 in the no-cues, blocked condition. 13

Supplementary Table S 8: Type III analysis of variance table with Kenward-Roger's method of the linear mixed effects model of Tcom/ External Torque of the first trial following a CoM change in the no-cues, blocked condition. 14

Supplementary Table S 9: Post-hoc t-tests of pairwise comparisons based on the LMM of Tcom/ External Torque of the first trial following a CoM change in the no-cues, blocked condition...14

Supplementary Table S 10: Type III analysis of variance table with Kenward-Roger's method of the linear mixed effects model of ΔCoP x GF/ External Torque of the first trial following a CoM change in the no-cues, blocked condition..14

Supplementary Table S 11: Post-hoc t-tests of pairwise comparisons based on the LMM of ΔCoP x GF/ External Torque of the first trial following a CoM change in the no-cues, blocked condition...14

Supplementary Table S 12: Type III analysis of variance table with Kenward-Roger's method of the linear mixed effects model of ΔFy x 0.5w/ External Torque of the first trial following a CoM change in the no-cues, blocked condition..14

Supplementary Table S 13: Post-hoc t-tests of pairwise comparisons based on the LMM of ΔFy x 0.5w/ External Torque of the first trial following a CoM change in the no-cues, blocked condition...14

Supplementary Table S 14: Type III analysis of variance table with Kenward-Roger's method of the linear mixed effects model of Tcom/ External Torque for the no-cues, pseudorandom condition...15

Supplementary Table S 15: Post-hoc t-tests of pairwise comparisons based on the LMM of Tcom/ External Torque for the no-cues, pseudorandom condition. 15

Supplementary Table S 16: Type III analysis of variance table with Kenward-Roger's method of the linear mixed effects model of ΔCoP x GF/ External Torque for the no-cues, pseudorandom condition. 15

Supplementary Table S 17: Post-hoc t-tests of pairwise comparisons based on the LMM of ΔCoP x GF/ External Torque for the no-cues, pseudorandom condition. 15

Supplementary Table S 18: Type III analysis of variance table with Kenward-Roger's method of the linear mixed effects model of ΔFy x 0.5w/ External Torque for the no-cues, pseudorandom condition. 16

Supplementary Table S 19: Post-hoc t-tests of pairwise comparisons based on the LMM of ΔFy x 0.5w/ External Torque for the no-cues, pseudorandom condition...............................16

Supplementary Table S 20: Type III analysis of variance table with Kenward-Roger's method of the linear mixed effects model of Tcom/ External Torque of trials 4-8 in the geometric cues, blocked condition. 16

Supplementary Table S 21: Post-hoc t-tests of pairwise comparisons based on the LMM of Tcom/ External Torque of trials 4-8 in the geometric cues, blocked condition. 16

Supplementary Table S 22: Type III analysis of variance table with Kenward-Roger's method of the linear mixed effects model of ΔCoP x GF/ External Torque of trials 4-8 in the geometric cues, blocked condition. 16

Supplementary Table S 23: Post-hoc t-tests of pairwise comparisons based on the LMM of ΔCoP x GF/ External Torque of trials 4-8 in the geometric cues, blocked condition. 17

Supplementary Table S 24: Type III analysis of variance table with Kenward-Roger's method of the linear mixed effects model of ΔFy x 0.5w/ External Torque of trials 4-8 in the geometric cues, blocked condition. 17

Supplementary Table S 25: Post-hoc t-tests of pairwise comparisons based on the LMM of ΔFy x 0.5w/ External Torque of trials 4-8 in the geometric cues, blocked condition. 17

Supplementary Table S 26: Type III analysis of variance table with Kenward-Roger's method of the linear mixed effects model of Tcom/ External Torque of the first trial following a CoM change in the geometric cues, blocked condition...17

Supplementary Table S 27: Post-hoc t-tests of pairwise comparisons based on the LMM of Tcom/ External Torque of the first trial following a CoM change in the geometric cues, blocked condition..17

Supplementary Table S 28: Type III analysis of variance table with Kenward-Roger's method of the linear mixed effects model of ΔCoP x GF/ External Torque of the first trial following a CoM change in the geometric cues, blocked condition. 18

Supplementary Table S 29: Post-hoc t-tests of pairwise comparisons based on the LMM of ΔCoP x GF/ External Torque of the first trial following a CoM change in the geometric cues, blocked condition..18

Supplementary Table S 30: Type III analysis of variance table with Kenward-Roger's method of the linear mixed effects model of ΔFy x 0.5w/ External Torque of the first trial following a CoM change in the geometric cues, blocked condition. 18

Supplementary Table S 31: Post-hoc t-tests of pairwise comparisons based on the LMM of ΔFy x 0.5w/ External Torque of the first trial following a CoM change in the geometric cues, blocked condition..18

Supplementary Table S 32: Type III analysis of variance table with Kenward-Roger's method of the linear mixed effects model of Tcom/ External Torque for the geometric cues, pseudorandom condition. 18

Supplementary Table S 33: Post-hoc t-tests of pairwise comparisons based on the LMM of Tcom/ External Torque for the geometric cues, pseudorandom condition............................18

Supplementary Table S 34: Type III analysis of variance table with Kenward-Roger's method of the linear mixed effects model of ΔCoP x GF/ External Torque for the geometric cues, pseudorandom condition. 19

Supplementary Table S 35: Post-hoc t-tests of pairwise comparisons based on the LMM of ΔCoP x GF/ External Torque for the geometric cues, pseudorandom condition. 19

Supplementary Table S 36: Type III analysis of variance table with Kenward-Roger's method of the linear mixed effects model of ΔFy x 0.5w/ External Torque for the geometric cues, pseudorandom condition. 19

Supplementary Table S 37: Post-hoc t-tests of pairwise comparisons based on the LMM of ΔFy x 0.5w/ External Torque for the geometric cues, pseudorandom condition. 19

Supplementary Table S 38: Type III analysis of variance table with Kenward-Roger's method of the linear mixed effects model of $\Delta CoP_{lift\ off}$ of trials 4-8 in the no-cues, blocked condition 20

Supplementary Table S 39: Post-hoc t-tests of pairwise comparisons between stroke patient- and hand-matched control groups based on the marginal means of the LMM of $\Delta CoP_{lift\ off}$ of trials 4-8 in the no-cues, blocked condition with Holm-Bonferroni correction for multiple testing applied 20

Supplementary Table S 40: Type III analysis of variance table with Kenward-Roger's method of the linear mixed effects model of $\Delta CoP_{lift\ off}$ for the no-cues, pseudorandom condition. 20

Supplementary Table S 41: Post-hoc t-tests of pairwise comparisons based on the LMM of $\Delta CoP_{lift\ off}$ for the no-cues, pseudorandom condition. 20

Supplementary Table S 42: Type III analysis of variance table with Kenward-Roger's method of the linear mixed effects model of $\Delta CoP_{lift\ off}$ of trials 4-8 in the geometric cues, blocked condition. 21

Supplementary Table S 43: Post-hoc t-tests of pairwise comparisons based on the LMM of $\Delta CoP_{lift\ off}$ of trials 4-8 in the geometric cues, blocked condition. 21

Supplementary Table S 44: Type III analysis of variance table with Kenward-Roger's method of the linear mixed effects model of $\Delta CoP_{lift\ off}$ for the geometric cues, pseudorandom condition. 21

Supplementary Table S 45: Post-hoc t-tests of pairwise comparisons based on the LMM of $\Delta CoP_{lift\ off}$ for the geometric cues, pseudorandom condition. 21

Supplementary Table S 46: Type III analysis of variance table with Kenward-Roger's method of the linear mixed effects model of $GF_{lift\ off}$ of trials 4-8 in the no-cues, blocked condition. 21

Supplementary Table S 47: Post-hoc t-tests of pairwise comparisons between stroke patient- and hand-matched control groups based on the marginal means of the LMM of $GF_{lift\ off}$ of trials 4-8 in the no-cues, blocked condition with Holm-Bonferroni correction for multiple testing applied. 21

Supplementary Table S 48: Type III analysis of variance table with Kenward-Roger's method of the linear mixed effects model of $GF_{lift\ off}$ for the no-cues, pseudorandom condition. 22

Supplementary Table S 49: Post-hoc t-tests of pairwise comparisons based on the LMM of $GF_{lift\ off}$ for the no-cues, pseudorandom condition. 22

Supplementary Table S 50: Type III analysis of variance table with Kenward-Roger's method of the linear mixed effects model of $GF_{lift\ off}$ of trials 4-8 in the geometric cues, blocked condition. 22

Supplementary Table S 51: Post-hoc t-tests of pairwise comparisons based on the LMM of $GF_{lift\ off}$ of trials 4-8 in the geometric cues, blocked condition. 22

Supplementary Table S 52: Type III analysis of variance table with Kenward-Roger's method of the linear mixed effects model of $GF_{lift\ off}$ for the geometric cues, pseudorandom condition. 23

Supplementary Table S 53: Post-hoc t-tests of pairwise comparisons based on the LMM of $GF_{lift\ off}$ for the geometric cues, pseudorandom condition. 23

3 Supplementary Figures

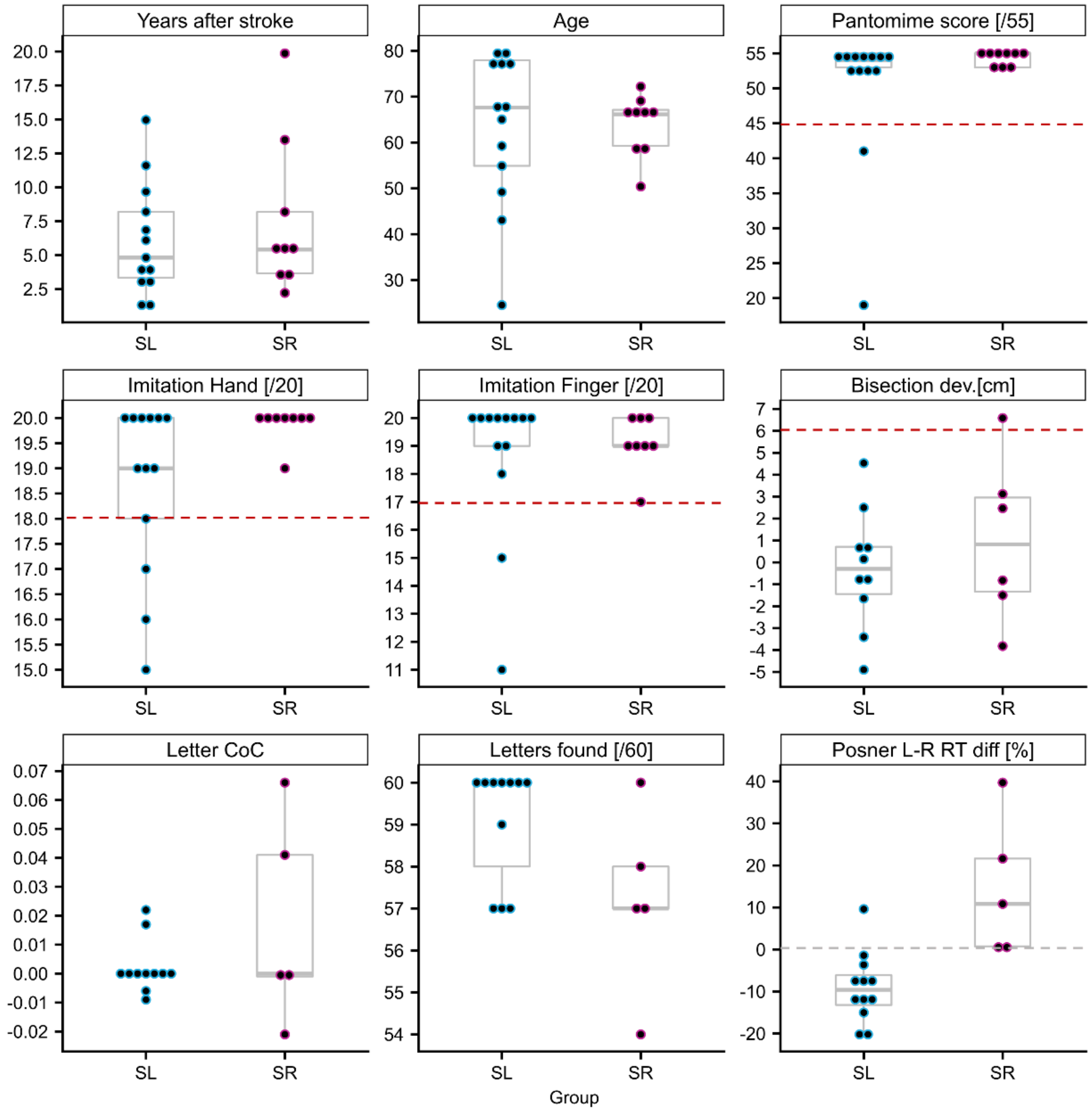

Supplementary Figure S 1: Demographics and clinical test results in left- and right hemisphere stroke patients. Red dashed lines delineate the boundaries of the normal range (inclusive). All participants performed within the normal range in the letter cancellation test (CoC ≤ 0.083).

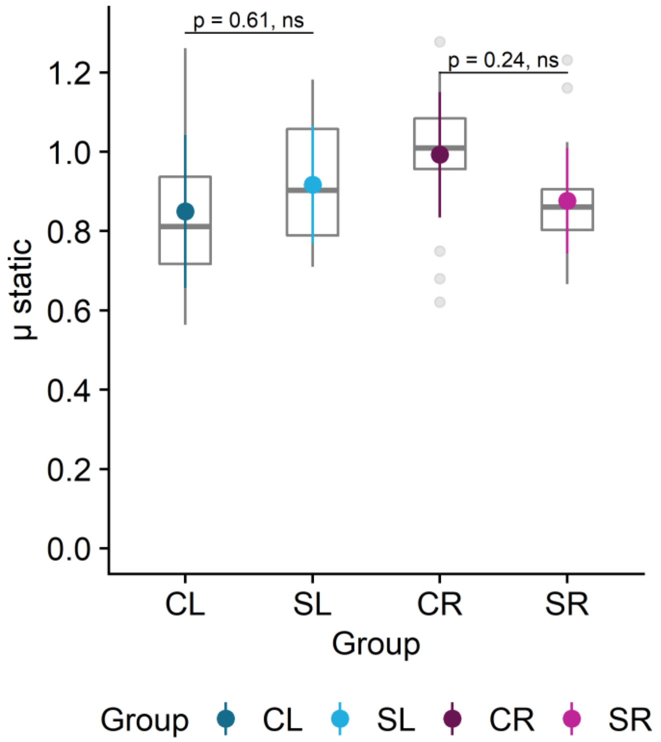

Supplementary Figure S 2: Coefficients of static friction as assessed by object slip trials. Holm-adjusted p-values of post-hoc t-tests of pairwise differences between controls and left- respectively right-hemispheric stroke patients. The groups' mean coefficients of friction did not significantly differ.

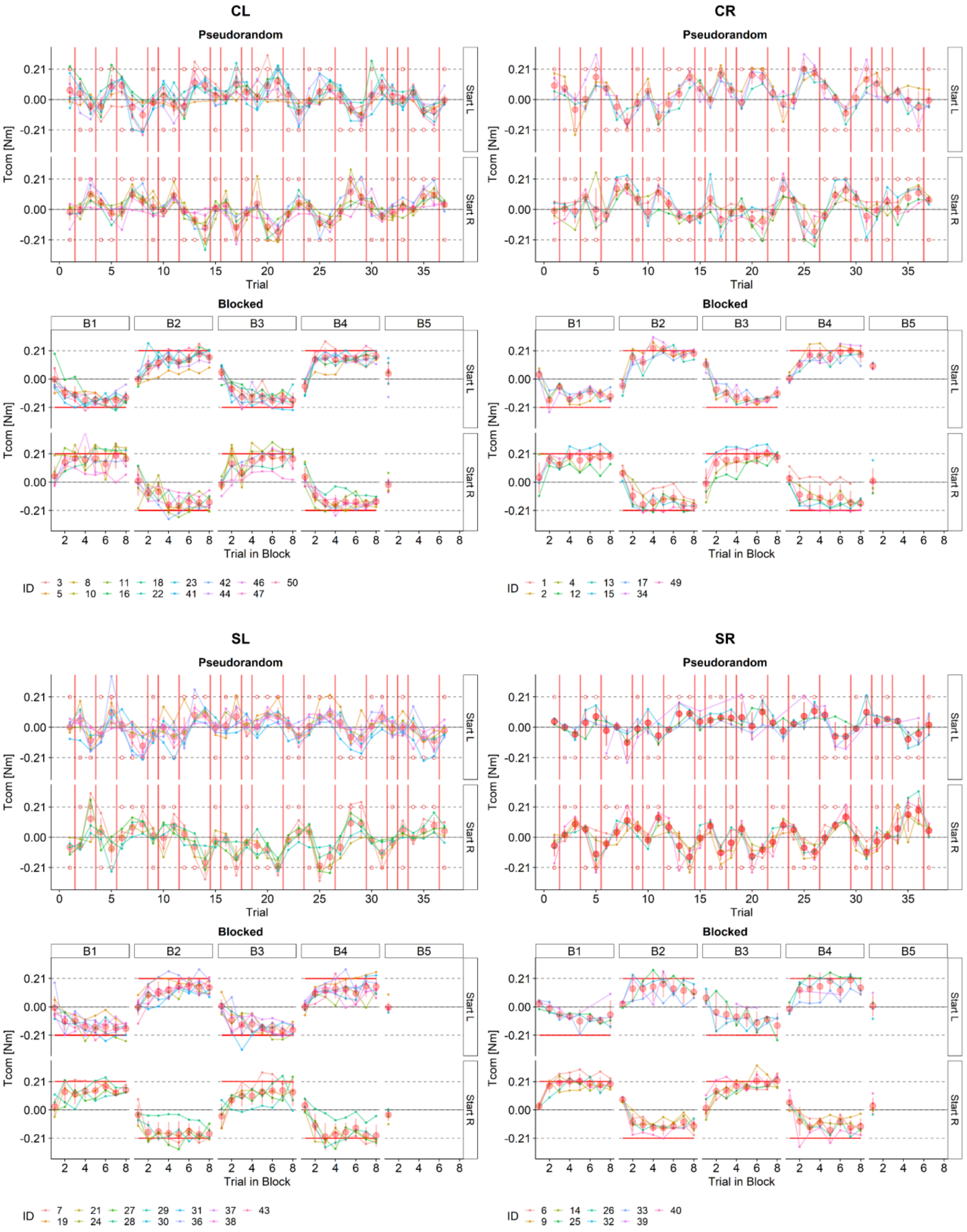

Supplementary Figure S 3: Individual and group averaged trajectories of Tcom of all trials in the no cues condition depicted for each group (CL: Controls, left hand, CR: Controls, right hand; SL: Left hemispheric stroke; SR: Right hemispheric stroke).

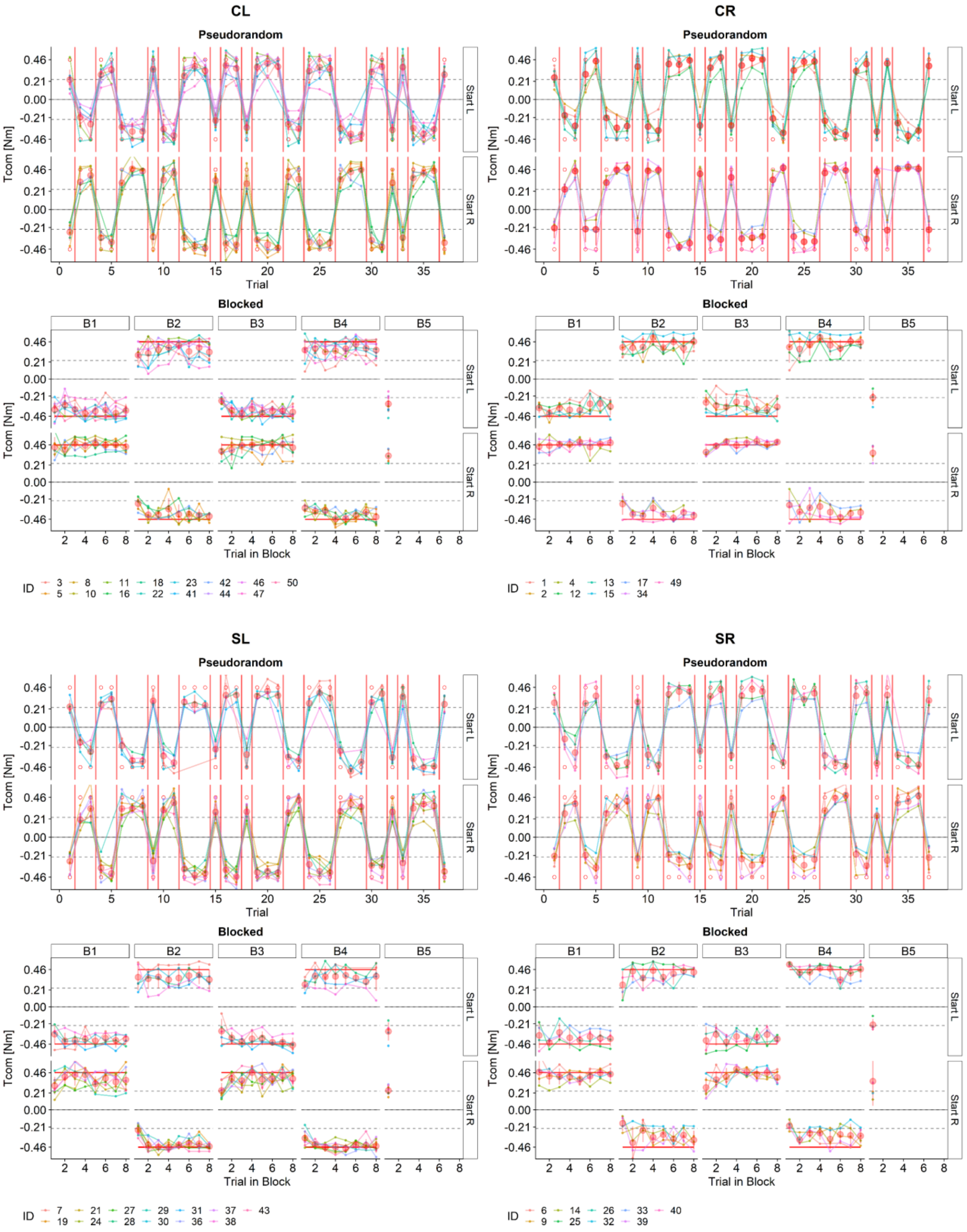

Supplementary Figure S 4: Individual and group averaged trajectories of Tcom of all trials in the geometric cues condition depicted for each group (CL: Controls, left hand, CR: Controls, right hand; SL: Left hemispheric stroke; SR: Right hemispheric stroke).

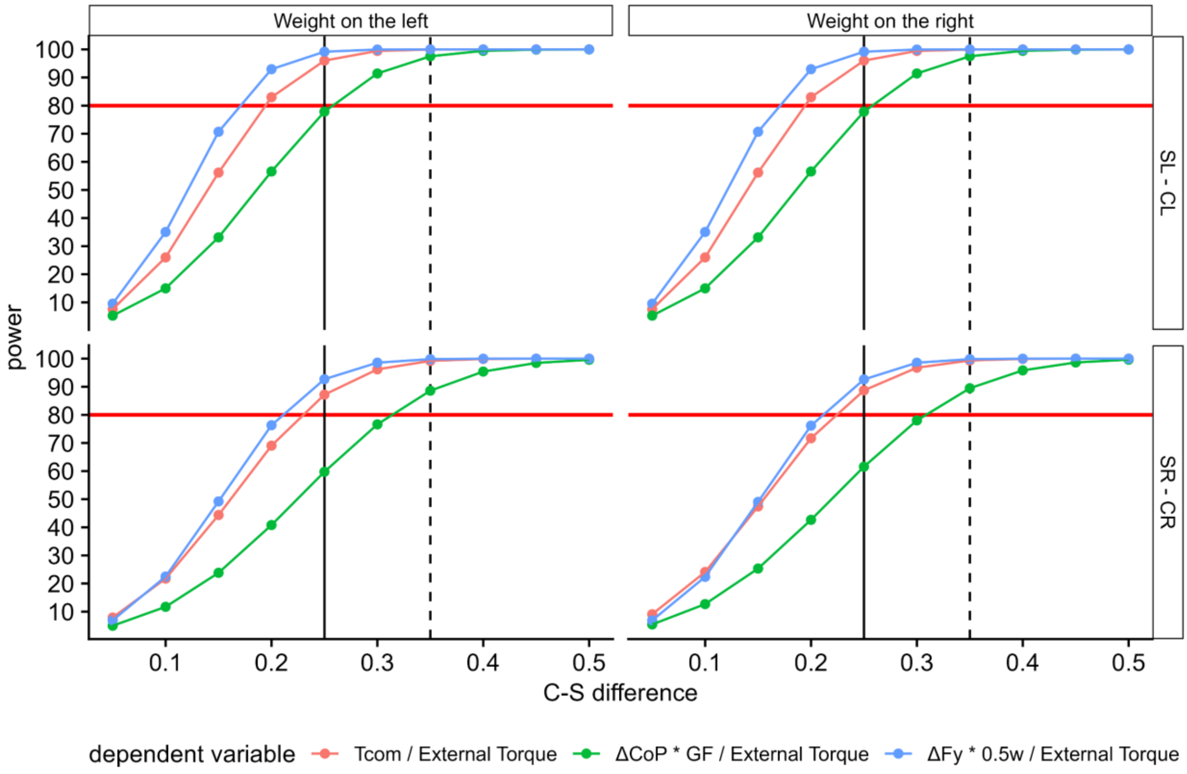

Supplementary Figure S 5: Results of a post-hoc sensitivity power analysis for the main outcome measures $\frac{\textbf{Tcom}}{\textbf{External Torque}}$**,** $\frac{\boldsymbol{\Delta}\textbf{Fy}*\textbf{w/2}}{\textbf{External Torque}}$ **and** $\frac{\boldsymbol{\Delta}\textbf{CoP} *\textbf{GF}}{\textbf{External Torque}}$ in **the no cues, blocked condition.** The outcome variables were repeatedly centered for each group (separately for both weight distributions) to yield group differences between the stroke and control groups between 0.05 and 0.5 in steps of 0.05. The alpha-level was set to 0.025. The figure depicts the power to detect a group difference between the respective control (‘C’) and stroke (‘S’) groups as a function of the group difference separately for the two group comparisons and weight distributions. We observed a higher power for comparisons between the ‘CL’ and ‘SL’ group than for comparisons between the ‘CR’ and ‘SR’ group. The lowest power was calculated for the outcome measure $\frac{\Delta \text{CoP} * \text{GF}}{\text{External Torque}}$. A power of >80% was observed for both group comparisons for $\frac{\text{Tcom}}{\text{External Torque}}$ and $\frac{\Delta \text{Fy}*\text{w/2}}{\text{External Torque}}$ group differences of 0.25, respectively 0.35 for $\frac{\Delta \text{CoP} * \text{GF}}{\text{External Torque}}$. Group comparisons between the ‘CL’ and ‘SL’ group were sufficiently powered (> 80%) to detect effects of 0.2, respectively 0.3 ($\frac{\Delta \text{CoP} * \text{GF}}{\text{External Torque}}$).

4 Supplementary Tables

Supplementary Table S 1: **Demographic and clinical information for all participating stroke patients.**

Clinical and radiological data were summarized from the available medical reports. **Abbreviations: YOS:** years since onset of stroke. **Type:** i = ischemic; h = hemorrhagic (ICH: intracerebral hemorrhage, SAH: subarachnoid hemorrhage); if two types are given, the first indicates the primary stroke type and the second a subsequent complication (e.g. i, h: ischemic stroke and post ischemic hemorrhage; **Side:** SL left hemispheric stroke, SR: right hemispheric stroke. **Cort./subc.:** cort. Cortex is affected, subc.: subcortical structures affected. **Location:** ACA: anterior cerebral artery, MCA: medial cerebral artery, PCA: posterior cerebral artery, part. = partial, inc. = incomplete, compl. = complete, terr. = territoritorial infarction in in the supply area of the respective artery, i.c. = internal capsule, put. = putamen, post. c. = post central, op. = opercular, f.b. =fronto-basal, ins. = insular, p. = parietal, t.p. = temporo-parietal, t.o. = temporo-occipital, p.o. = parietooccipital, cort. = cortex, pall. = pallidum, med. l. = medullary layer, BG = basal ganglia, periv. = periventricular, , th. = thalamus, **Letter**: Letter cancellation test, **CoC**: Center of Cancellation, **Posner:** Posner reaction time test, median reaction time for stimuli on left (L) and right (R). **Protocol:** Experimental Protocol indicating order of visual (V) and no-cue (N) conditions as well as first side of CoM in the first trial of the respective experimental condition (L: left, R: right). **Symptoms described in medical reports:** Presence of documented symptoms are denoted with 1.

ID	Gender	Age	YOS	Stroke Lesion				mRS	Pantomime		Imitation		Bisect. Dev.	Letter		Posner		Protocol	Max GF	Report of symptoms in medical reports				
				Type	Side	cort./ subc.	Location:		Correct items [/20]	Score [/55]	Hand [/20]	Finger [/20]	[mm]	CoC	found [/60]	L [ms]	R [ms]		[N]	Paresis	Sens. loss	Aphasia	Neglect	Anopsia
7	f	67.9	4.2	i	SL	subc.	i.c., put.	2	19	54	20	20	0.15	0.000	60	467.5	573.5	VLNR	43.4	1	1	0	0	0
19	m	59.2	8.2	i	SL	cort., subc.	Part. MCA terr. (post.c.-,op.-,f.b.-ins.cort., BG, pall., med. l.)	2	20	55	19	19	2.50	0.000	60	329.5	383.0	VRNL	98.5	1	1	1	0	1
21	f	65.0	15.0	h, i	SL	subc.	Part. MCA terr. (BG, med. l.)	3	20	55	20	20	0.76	-0.009	59	558.0	566.0	NRVR	34.7	1	0	1	0	0
24	f	67.6	3.7	i	SL	cort., subc.	Inc. MCA terr. (fr., p., t.p.-lobes, periv., med. l., i.c.)	3	11	41	16	16	NA	NA	NA	943.0	978.0	NLVR	57.7	1	1	1	0	0
27	m	77.9	6.9	i	SL	cort., subc.	Inc. MCA terr.	4	3	19	15	15	-3.41	0.017	57	677.5	828.5	VRNR	59.0	1	1	1	0	1
28	f	43.1	9.7	i	SL	cort., subc.	Part. MCA terr. (op. cort., BG)	2	20	53	20	20	-1.65	NA	NA	312.5	335.5	NRVL	51.0	1	1	1	0	0
29	m	78.0	6.1	i, h	SL	cort., subc.	Part. MCA terr. (t.o. lobe (h))	1	19	54	19	19	4.53	-0.006	57	561.5	628.0	VRNR	72.4	1	0	1	1	1
30	m	49.2	2.7	i	SL	cort., subc.	MCA terr.	2	18	53	20	20	NA	0.000	60	382.5	431.0	NLVL	69.2	1	0	1	1	0
31	m	79.1	3.4	i	SL	cort., subc.	Part. MCA terr. (p.-, t. lobes , BG)	3	19	54	20	20	-0.82	0.022	57	461.0	499.5	VLNL	94.8	1	1	1	0	1
36	m	54.9	1.5	il	SL	cort., subc.	Part. PCA terr.	2	20	55	20	20	0.58	0.000	60	485.0	520.0	VRNL	97.2	0	0	0	0	0
37	f	76.3	4.8	i	SL	cort., subc.	Inc. MCA terr.	3	20	55	19	19	-4.90	0.000	60	486.5	552.0	NLVR	55.2	1	0	1	0	0
38	m	79.8	1.1	i	SL	subc.	Part. MCA terr (i.c.)	2	18	53	18	18	-0.74	0.000	60	741.5	673.5	VLNL	83.0	1	0	0	0	0
43	f	24.5	11.6	h, i	SL	cort., subc.	Part. MCA terr.	2	17	52	17	17	NA	0.000	60	NA	NA	NLVR	63.0	1	1	1	0	0
6	f	59.3	3.7	h	SR	subc.	ICB Th, SAB fr., p.o. lobe	2	20	55	NA	NA	NA	NA	NA	NA	NA	NRVR	79.0	1	0	1	1	0
9	f	50.4	13.5	i	SR	cort., subc.	Inc. MCA terr.	2	20	55	20	20	6.59	0.000	60	538.0	433.0	VRNR	53.6	1	1	0	0	1
14	m	66.0	5.4	i	SR	subc.	Pons	1	20	55	20	20	NA	NA	NA	405.0	402.0	NRVR	93.3	1	0	0	0	0
25	m	72.2	2.2	i	SR	cort., subc.	Inc. MCA terr.	2	18	53	20	20	-0.82	0.041	57	NA	NA	NLVL	67.4	1	0	1	0	0
26	f	69.1	5.2	h	SR	subc.	t.p. lobe	3	20	55	20	20	-3.82	NA	NA	739.5	494.5	VLNR	37.8	1	1	0	0	1
32	m	66.4	3.5	i	SR	cort., subc.	f.-, p.o. lobes	1	20	55	20	20	-1.50	0.066	54	460.5	459.0	VRNL	62.4	1	0	0	0	0
33	f	66.1	5.8	I	SR	cort., subc.	Compl. MCA, PCA-, inc. ACA terr.	4	18	53	20	20	NA	NA	NA	NA	NA	NLVL	63.3	1	1	1	0	1
39	f	67.1	19.9	h	SR	cort., subc.	Compl. MCA terr.	4	18	53	19	19	3.12	-0.001	57	NA	NA	VRNL	60.1	1	0	0	0	0
40	m	58.0	8.2	h	SR	subc.	BG, medull..l,	1	20	55	20	20	2.47	-0.021	58	428.0	384.0	VLNR	76.9	1	0	0	0	0

Supplementary Table S 2: **Type III analysis of variance table with Kenward-Roger's method of the linear mixed effects model of Tcom/ External Torque of trials 4-8 in the no-cues, blocked condition**

	Sum Sq	Mean Sq	NumDF	DenDF	F value	Pr(>F)
**Ext. Torque**	3.682	3.682	1	858.112	57.531	0.000
**Group**	0.204	0.068	3	42.076	1.061	0.376
**Ext. Torque x Group**	6.373	2.124	3	858.099	33.191	0.000

Supplementary Table S 3: **Post-hoc t-tests of pairwise comparisons between stroke patient- and hand-matched control groups based on the marginal means of the LMM of Tcom/ External Torque of trials 4-8 in the no-cues, blocked condition with Holm-Bonferroni correction for multiple testing applied**

contrast	external torque	estimate	SE	df	t.ratio	conf.int	partial eta squared	Cohen's f	p
**SL - CL**	-0.21	0.05	0.06	54.21	0.83	[-0.09 ; 0.19]	0.01	0.11	0.408
**SR - CR**	-0.21	-0.16	0.08	55.34	-2.15	[-0.34 ; 0.01]	0.08	0.29	0.071
**SL - CL**	0.21	-0.11	0.06	54.21	-1.75	[-0.25 ; 0.03]	0.05	0.24	0.170
**SR - CR**	0.21	-0.07	0.08	55.44	-0.97	[-0.25 ; 0.1]	0.02	0.13	0.338

Supplementary Table S 4: **Type III analysis of variance table with Kenward-Roger's method of the linear mixed effects model of ∆CoP x GF/ External Torque of trials 4-8 in the no-cues, blocked condition**

	Sum Sq	Mean Sq	NumDF	DenDF	F value	Pr(>F)
**Ext. Torque**	0.307	0.307	1	858.194	1.624	0.203
**Group**	0.299	0.100	3	42.089	0.529	0.665
**Ext. Torque x Group**	30.408	10.136	3	858.174	53.675	0.000

Supplementary Table S 5: Post-hoc t-tests of pairwise comparisons based on the LMM of ∆CoP x GF/ External Torque of trials 4-8 in the no-cues, blocked condition.

contrast	external torque	estimate	SE	df	t.ratio	conf.int	partial eta squared	Cohen's f	p
**SL - CL**	-0.21	0.33	0.08	65.45	4.10	[0.15 ; 0.52]	0.20	0.51	0.000
**SR - CR**	-0.21	-0.22	0.10	67.44	-2.16	[-0.46 ; 0.01]	0.06	0.26	0.035
**SL - CL**	0.21	-0.37	0.08	65.45	-4.59	[-0.56 ; -0.19]	0.24	0.57	0.000
**SR - CR**	0.21	0.03	0.10	67.53	0.28	[-0.21 ; 0.26]	0.00	0.03	0.779

Supplementary Table S 6: Type III analysis of variance table with Kenward-Roger's method of the linear mixed effects model of ∆Fy x 0.5w/ External Torque of trials 4-8 in the no-cues, blocked condition

	Sum Sq	Mean Sq	NumDF	DenDF	F value	Pr(>F)
**Ext. Torque**	6.129	6.129	1	858.367	43.778	0.000
**Group**	0.077	0.026	3	42.041	0.184	0.907
**Ext. Torque x Group**	12.286	4.095	3	858.338	29.254	0.000

Supplementary Table S 7: Post-hoc t-tests of pairwise comparisons based on the LMM of ∆Fy x 0.5w/ External Torque of trials 4-8 in the no-cues, blocked condition.

contrast	external torque	estimate	SE	df	t.ratio	conf.int	partial eta squared	Cohen's f	p
**SL - CL**	-0.21	-0.28	0.05	98.18	-5.33	[-0.4 ; -0.16]	0.22	0.54	0.000
**SR - CR**	-0.21	0.06	0.07	102.06	0.90	[-0.09 ; 0.21]	0.01	0.09	0.372
**SL - CL**	0.21	0.27	0.05	98.18	5.03	[0.15 ; 0.39]	0.20	0.51	0.000

**SR - CR**	0.21	-0.10	0.07	101.76	-1.45	[-0.25 ; 0.06]	0.02	0.14	0.149

Supplementary Table S 8: Type III analysis of variance table with Kenward-Roger's method of the linear mixed effects model of Tcom/ External Torque of the first trial following a CoM change in the no-cues, blocked condition.

	Sum Sq	Mean Sq	NumDF	DenDF	F value	Pr(>F)
**Ext. Torque**	3.682	3.682	1	858.112	57.531	0.000
**Group**	0.204	0.068	3	42.076	1.061	0.376
**Ext. Torque x Group**	6.373	2.124	3	858.099	33.191	0.000

Supplementary Table S 9: Post-hoc t-tests of pairwise comparisons based on the LMM of Tcom/ External Torque of the first trial following a CoM change in the no-cues, blocked condition.

contrast	external torque	estimate	SE	df	t.ratio	conf.int	partial eta squared	Cohen's f	p
**SL - CL**	-0.21	0.17	0.08	95.45	2.03	[-0.02 ; 0.36]	0.04	0.21	0.091
**SR - CR**	-0.21	0.09	0.11	97.80	0.86	[-0.15 ; 0.33]	0.01	0.09	0.392
**SL - CL**	0.21	0.04	0.08	95.45	0.45	[-0.15 ; 0.23]	0.00	0.05	0.653
**SR - CR**	0.21	0.12	0.11	97.80	1.13	[-0.12 ; 0.36]	0.01	0.11	0.522

Supplementary Table S 10: Type III analysis of variance table with Kenward-Roger's method of the linear mixed effects model of ∆CoP x GF/ External Torque of the first trial following a CoM change in the no-cues, blocked condition.

	Sum Sq	Mean Sq	NumDF	DenDF	F value	Pr(>F)
**Ext. Torque**	0.307	0.307	1	858.194	1.624	0.203
**Group**	0.299	0.100	3	42.089	0.529	0.665
**Ext. Torque x Group**	30.408	10.136	3	858.174	53.675	0.000

Supplementary Table S 11: Post-hoc t-tests of pairwise comparisons based on the LMM of ∆CoP x GF/ External Torque of the first trial following a CoM change in the no-cues, blocked condition.

contrast	external torque	estimate	SE	df	t.ratio	conf.int	partial eta squared	Cohen's f	p
**SL - CL**	-0.21	0.33	0.12	126.20	2.81	[0.06 ; 0.59]	0.06	0.25	0.012
**SR - CR**	-0.21	-0.01	0.15	128.21	-0.04	[-0.34 ; 0.33]	0.00	0.00	0.972
**SL - CL**	0.21	-0.19	0.12	126.20	-1.64	[-0.46 ; 0.07]	0.02	0.15	0.207
**SR - CR**	0.21	0.24	0.15	128.21	1.60	[-0.1 ; 0.57]	0.02	0.14	0.207

Supplementary Table S 12: Type III analysis of variance table with Kenward-Roger's method of the linear mixed effects model of ∆Fy x 0.5w/ External Torque of the first trial following a CoM change in the no-cues, blocked condition.

	Sum Sq	Mean Sq	NumDF	DenDF	F value	Pr(>F)
**Ext. Torque**	6.129	6.129	1	858.367	43.778	0.000
**Group**	0.077	0.026	3	42.041	0.184	0.907
**Ext. Torque x Group**	12.286	4.095	3	858.338	29.254	0.000

Supplementary Table S 13: Post-hoc t-tests of pairwise comparisons based on the LMM of ∆Fy x 0.5w/ External Torque of the first trial following a CoM change in the no-cues, blocked condition.

contrast	external torque	estimate	SE	df	t.ratio	conf.int	partial eta squared	Cohen's f	p
**SL - CL**	-0.21	-0.16	0.10	126.20	-1.55	[-0.39 ; 0.07]	0.02	0.14	0.246
**SR - CR**	-0.21	0.10	0.13	128.21	0.76	[-0.19 ; 0.39]	0.00	0.07	0.449

**SL - CL**	0.21	0.23	0.10	126.20	2.26	[0 ; 0.46]	0.04	0.20	0.051
**SR - CR**	0.21	-0.12	0.13	128.21	-0.90	[-0.41 ; 0.18]	0.01	0.08	0.368

Supplementary Table S 14: **Type III analysis of variance table with Kenward-Roger's method of the linear mixed effects model of Tcom/ External Torque for the no-cues, pseudorandom condition.**

	Sum Sq	Mean Sq	NumDF	DenDF	F value	Pr(>F)
**Ext. Torque**	4.681	4.681	1	1568.656	46.116	0.000
**CoM action**	116.831	116.831	1	1569.860	1151.016	0.000
**Group**	0.193	0.064	3	42.065	0.635	0.597
**Ext. Torque x CoM action**	0.184	0.184	1	1568.698	1.808	0.179
**Ext. Torque x Group**	5.497	1.832	3	1568.611	18.051	0.000
**CoM action x Group**	1.165	0.388	3	1569.623	3.827	0.010
**Ext. Torque x CoM action x Group**	0.410	0.137	3	1568.628	1.346	0.258

Supplementary Table S 15: Post-hoc t-tests of pairwise comparisons based on the LMM of Tcom/ External Torque for the no-cues, pseudorandom condition.

contrast	external torque	CoM action	estimate	SE	df	t.ratio	conf.int	partial eta squared	Cohen's f	p
**SL - CL**	-0.21	CoM retained	0.08	0.05	236.90	1.70	[-0.03 ; 0.18]	0.01	0.11	0.183
**SL - CL**	-0.21	CoM inverted	0.10	0.05	236.90	2.28	[0 ; 0.21]	0.01	0.11	0.046
**SR - CR**	-0.21	CoM retained	0.08	0.06	246.45	1.44	[-0.05 ; 0.21]	0.01	0.09	0.183
**SR - CR**	-0.21	CoM inverted	0.11	0.06	250.10	1.91	[-0.02 ; 0.24]	0.01	0.09	0.057
**SL - CL**	0.21	CoM retained	-0.05	0.05	238.06	-1.10	[-0.15 ; 0.05]	0.01	0.07	0.271
**SL - CL**	0.21	CoM inverted	-0.05	0.05	240.46	-1.11	[-0.15 ; 0.05]	0.01	0.07	0.267
**SR - CR**	0.21	CoM retained	-0.13	0.06	246.74	-2.33	[-0.26 ; 0]	0.02	0.15	0.041
**SR - CR**	0.21	CoM inverted	0.09	0.06	252.86	1.57	[-0.04 ; 0.22]	0.02	0.15	0.238

Supplementary Table S 16: Type III analysis of variance table with Kenward-Roger's method of the linear mixed effects model of ∆CoP x GF/ External Torque for the no-cues, pseudorandom condition.

	Sum Sq	Mean Sq	NumDF	DenDF	F value	Pr(>F)
**Ext. Torque**	5.761	5.761	1	1569.053	29.700	0.000
**CoM action**	65.335	65.335	1	1570.488	336.807	0.000
**Group**	0.276	0.092	3	41.937	0.474	0.702
**Ext. Torque x CoM action**	0.073	0.073	1	1569.093	0.378	0.539
**Ext. Torque x Group**	17.578	5.859	3	1569.004	30.206	0.000
**CoM action x Group**	0.862	0.287	3	1570.202	1.482	0.218
**Ext. Torque x CoM action x Group**	0.092	0.031	3	1569.003	0.159	0.924

Supplementary Table S 17: Post-hoc t-tests of pairwise comparisons based on the LMM of ∆CoP x GF/ External Torque for the no-cues, pseudorandom condition.

contrast	external torque	CoM action	estimate	SE	df	t.ratio	conf.int	partial eta squared	Cohen's f	p
**SL - CL**	-0.21	CoM retained	0.24	0.06	528.96	4.28	[0.11 ; 0.36]	0.03	0.19	0.000
**SL - CL**	-0.21	CoM inverted	0.23	0.06	528.96	4.22	[0.11 ; 0.36]	0.03	0.19	0.000
**SR - CR**	-0.21	CoM retained	-0.01	0.07	545.22	-0.09	[-0.16 ; 0.15]	0.00	0.00	0.932
**SR - CR**	-0.21	CoM inverted	0.10	0.07	550.00	1.42	[-0.06 ; 0.26]	0.00	0.00	0.157
**SL - CL**	0.21	CoM retained	-0.21	0.06	531.42	-3.72	[-0.33 ; -0.08]	0.03	0.16	0.000
**SL - CL**	0.21	CoM inverted	-0.27	0.06	536.54	-4.79	[-0.39 ; -0.14]	0.03	0.16	0.000
**SR - CR**	0.21	CoM retained	-0.05	0.07	546.01	-0.65	[-0.2 ; 0.11]	0.00	0.03	0.517

**SR - CR**	0.21	CoM inverted	0.12	0.07	547.40	1.67	[-0.04 ; 0.28]	0.00	0.03	0.096

Supplementary Table S 18: Type III analysis of variance table with Kenward-Roger's method of the linear mixed effects model of ∆Fy x 0.5w/ External Torque for the no-cues, pseudorandom condition.

	Sum Sq	Mean Sq	NumDF	DenDF	F value	Pr(>F)
**Ext. Torque**	20.820	20.820	1	1569.053	142.426	0.000
**CoM action**	7.443	7.443	1	1570.488	50.917	0.000
**Group**	0.163	0.054	3	41.937	0.372	0.773
**Ext. Torque x CoM action**	0.025	0.025	1	1569.093	0.168	0.682
**Ext. Torque x Group**	7.395	2.465	3	1569.004	16.862	0.000
**CoM action x Group**	0.329	0.110	3	1570.202	0.751	0.522
**Ext. Torque x CoM action x Group**	0.265	0.088	3	1569.003	0.604	0.612

Supplementary Table S 19: Post-hoc t-tests of pairwise comparisons based on the LMM of ∆Fy x 0.5w/ External Torque for the no-cues, pseudorandom condition.

contrast	external torque	CoM action	estimate	SE	df	t.ratio	conf.int	partial eta squared	Cohen's f	p
**SL - CL**	-0.21	CoM retained	-0.16	0.05	528.96	-3.35	[-0.27 ; -0.05]	0.02	0.15	0.002
**SL - CL**	-0.21	CoM inverted	-0.13	0.05	528.96	-2.72	[-0.24 ; -0.02]	0.02	0.15	0.014
**SR - CR**	-0.21	CoM retained	0.09	0.06	545.22	1.43	[-0.05 ; 0.22]	0.00	0.06	0.154
**SR - CR**	-0.21	CoM inverted	0.01	0.06	550.00	0.12	[-0.13 ; 0.15]	0.00	0.06	0.905
**SL - CL**	0.21	CoM retained	0.16	0.05	531.42	3.25	[0.05 ; 0.27]	0.02	0.14	0.002
**SL - CL**	0.21	CoM inverted	0.22	0.05	536.54	4.47	[0.11 ; 0.33]	0.02	0.14	0.000
**SR - CR**	0.21	CoM retained	-0.09	0.06	546.01	-1.45	[-0.23 ; 0.05]	0.00	0.06	0.147
**SR - CR**	0.21	CoM inverted	-0.03	0.06	547.40	-0.49	[-0.17 ; 0.11]	0.00	0.06	0.622

Supplementary Table S 20: Type III analysis of variance table with Kenward-Roger's method of the linear mixed effects model of Tcom/ External Torque of trials 4-8 in the geometric cues, blocked condition.

	Sum Sq	Mean Sq	NumDF	DenDF	F value	Pr(>F)
**Ext. Torque**	1.153	1.153	1	859.272	38.471	0.000
**Group**	0.015	0.005	3	42.009	0.161	0.922
**Ext. Torque x Group**	3.791	1.264	3	859.260	42.146	0.000

Supplementary Table S 21: Post-hoc t-tests of pairwise comparisons based on the LMM of Tcom/ External Torque of trials 4-8 in the geometric cues, blocked condition.

contrast	external torque	estimate	SE	df	t.ratio	conf.int	partial eta squared	Cohen's f	p
**SL - CL**	-0.46	0.06	0.04	52.66	1.40	[-0.04 ; 0.17]	0.04	0.19	0.333
**SR - CR**	-0.46	-0.01	0.06	53.00	-0.20	[-0.14 ; 0.12]	0.00	0.03	0.839
**SL - CL**	0.46	-0.06	0.04	53.10	-1.35	[-0.16 ; 0.04]	0.03	0.18	0.367
**SR - CR**	0.46	-0.05	0.06	52.87	-0.97	[-0.18 ; 0.07]	0.02	0.13	0.367

Supplementary Table S 22: Type III analysis of variance table with Kenward-Roger's method of the linear mixed effects model of ∆CoP x GF/ External Torque of trials 4-8 in the geometric cues, blocked condition.

	Sum Sq	Mean Sq	NumDF	DenDF	F value	Pr(>F)
**Ext. Torque**	0.507	0.507	1	859.233	10.439	0.001
**Group**	0.050	0.017	3	42.008	0.345	0.793
**Ext. Torque x Group**	7.501	2.500	3	859.223	51.472	0.000

Supplementary Table S 23: Post-hoc t-tests of pairwise comparisons based on the LMM of ∆CoP x GF/ External Torque of trials 4-8 in the geometric cues, blocked condition.

contrast	external torque	estimate	SE	df	t.ratio	conf.int	partial eta squared	Cohen's f	p
**SL - CL**	-0.46	0.12	0.06	51.03	1.93	[-0.02 ; 0.26]	0.07	0.27	0.118
**SR - CR**	-0.46	0.05	0.08	51.31	0.67	[-0.12 ; 0.23]	0.01	0.09	0.504
**SL - CL**	0.46	-0.09	0.06	51.40	-1.43	[-0.23 ; 0.05]	0.04	0.20	0.318
**SR - CR**	0.46	0.02	0.08	51.20	0.31	[-0.15 ; 0.2]	0.00	0.04	0.759

Supplementary Table S 24: Type III analysis of variance table with Kenward-Roger's method of the linear mixed effects model of ∆Fy x 0.5w/ External Torque of trials 4-8 in the geometric cues, blocked condition.

	Sum Sq	Mean Sq	NumDF	DenDF	F value	Pr(>F)
ExtTorque_F	3.189	3.189	1	859.323	117.067	0.000
Group	0.092	0.031	3	42.011	1.131	0.347
ExtTorque_F:Group	0.673	0.224	3	859.308	8.237	0.000

	Sum Sq	Mean Sq	NumDF	DenDF	F value	Pr(>F)
**Ext. Torque**	3.189	3.189	1	859.323	117.067	0.000
**Group**	0.092	0.031	3	42.011	1.131	0.347
**Ext. Torque x Group**	0.673	0.224	3	859.308	8.237	0.000

Supplementary Table S 25: Post-hoc t-tests of pairwise comparisons based on the LMM of ∆Fy x 0.5w/ External Torque of trials 4-8 in the geometric cues, blocked condition.

contrast	external torque	estimate	SE	df	t.ratio	conf.int	partial eta squared	Cohen's f	p
**SL - CL**	-0.46	-0.06	0.04	54.81	-1.40	[-0.15 ; 0.04]	0.03	0.19	0.332
**SR - CR**	-0.46	-0.06	0.05	55.21	-1.27	[-0.18 ; 0.05]	0.03	0.17	0.332
**SL - CL**	0.46	0.03	0.04	55.34	0.68	[-0.06 ; 0.12]	0.01	0.09	0.497
**SR - CR**	0.46	-0.08	0.05	55.05	-1.57	[-0.19 ; 0.04]	0.04	0.21	0.243

Supplementary Table S 26: Type III analysis of variance table with Kenward-Roger's method of the linear mixed effects model of Tcom/ External Torque of the first trial following a CoM change in the geometric cues, blocked condition.

	Sum Sq	Mean Sq	NumDF	DenDF	F value	Pr(>F)
**Ext. Torque**	0.583	0.583	1	133.224	10.122	0.002
**Group**	0.103	0.034	3	41.988	0.597	0.621
**Ext. Torque x Group**	0.592	0.197	3	132.746	3.426	0.019

Supplementary Table S 27: Post-hoc t-tests of pairwise comparisons based on the LMM of Tcom/ External Torque of the first trial following a CoM change in the geometric cues, blocked condition.

contrast	external torque	estimate	SE	df	t.ratio	conf.int	partial eta squared	Cohen's f	p
**SL - CL**	-0.46	0.02	0.08	90.96	0.28	[-0.15 ; 0.19]	0.00	0.03	1.000
**SR - CR**	-0.46	-0.02	0.10	94.27	-0.22	[-0.24 ; 0.2]	0.00	0.02	1.000
**SL - CL**	0.46	-0.15	0.08	92.30	-1.92	[-0.32 ; 0.03]	0.04	0.20	0.116
**SR - CR**	0.46	-0.09	0.10	93.42	-0.91	[-0.3 ; 0.13]	0.01	0.09	0.366

Supplementary Table S 28: Type III analysis of variance table with Kenward-Roger's method of the linear mixed effects model of ∆CoP x GF/ External Torque of the first trial following a CoM change in the geometric cues, blocked condition.

	Sum Sq	Mean Sq	NumDF	DenDF	F value	Pr(>F)
**Ext. Torque**	0.019	0.019	1	133.314	0.231	0.632
**Group**	0.180	0.060	3	41.974	0.732	0.539
**Ext. Torque x Group**	0.922	0.307	3	132.828	3.738	0.013

Supplementary Table S 29: Post-hoc t-tests of pairwise comparisons based on the LMM of ∆CoP x GF/ External Torque of the first trial following a CoM change in the geometric cues, blocked condition.

contrast	external torque	estimate	SE	df	t.ratio	conf.int	partial eta squared	Cohen's f	p
**SL - CL**	-0.46	0.02	0.09	94.53	0.28	[-0.18 ; 0.23]	0.00	0.03	1.000
**SR - CR**	-0.46	0.02	0.11	97.60	0.19	[-0.24 ; 0.28]	0.00	0.02	1.000
**SL - CL**	0.46	-0.23	0.09	95.89	-2.60	[-0.44 ; -0.03]	0.07	0.27	0.021
**SR - CR**	0.46	-0.01	0.11	97.02	-0.13	[-0.27 ; 0.24]	0.00	0.01	0.897

Supplementary Table S 30: Type III analysis of variance table with Kenward-Roger's method of the linear mixed effects model of ∆Fy x 0.5w/ External Torque of the first trial following a CoM change in the geometric cues, blocked condition.

	Sum Sq	Mean Sq	NumDF	DenDF	F value	Pr(>F)
**Ext. Torque**	0.811	0.811	1	133.810	21.036	0.000
**Group**	0.118	0.039	3	41.831	1.016	0.395
**Ext. Torque x Group**	0.063	0.021	3	133.308	0.540	0.656

Supplementary Table S 31: Post-hoc t-tests of pairwise comparisons based on the LMM of ∆Fy x 0.5w/ External Torque of the first trial following a CoM change in the geometric cues, blocked condition.

contrast	external torque	estimate	SE	df	t.ratio	conf.int	partial eta squared	Cohen's f	p
**SL - CL**	-0.46	0.00	0.05	124.28	-0.07	[-0.12 ; 0.12]	0.00	0.01	1.000
**SR - CR**	-0.46	-0.04	0.07	124.28	-0.61	[-0.19 ; 0.11]	0.00	0.05	1.000
**SL - CL**	0.46	0.08	0.05	125.36	1.58	[-0.04 ; 0.2]	0.02	0.14	0.233
**SR - CR**	0.46	-0.07	0.07	126.28	-1.11	[-0.22 ; 0.08]	0.01	0.10	0.267

Supplementary Table S 32: Type III analysis of variance table with Kenward-Roger's method of the linear mixed effects model of Tcom/ External Torque for the geometric cues, pseudorandom condition.

	Sum Sq	Mean Sq	NumDF	DenDF	F value	Pr(>F)
**Ext. Torque**	2.390	2.390	1	1568.212	56.116	0.000
**CoM action**	10.663	10.663	1	1568.193	250.314	0.000
**Group**	0.049	0.016	3	41.980	0.383	0.766
**Ext. Torque x CoM action**	0.012	0.012	1	1568.090	0.292	0.589
**Ext. Torque x Group**	4.910	1.637	3	1568.230	38.419	0.000
**CoM action x Group**	0.239	0.080	3	1568.211	1.870	0.133
**Ext. Torque x CoM action x Group**	0.041	0.014	3	1568.092	0.322	0.809

Supplementary Table S 33: Post-hoc t-tests of pairwise comparisons based on the LMM of Tcom/ External Torque for the geometric cues, pseudorandom condition.

contrast	external torque	CoM action	estimate	SE	df	t.ratio	conf.int	partial eta squared	Cohen's f	p

**SL - CL**	-0.46	CoM retained	0.04	0.05	63.76	0.83	[-0.08 ; 0.16]	0.01	0.10	0.820
**SL - CL**	-0.46	CoM inverted	0.00	0.05	63.42	0.09	[-0.12 ; 0.12]	0.01	0.10	1.000
**SR - CR**	-0.46	CoM retained	-0.01	0.07	63.29	-0.20	[-0.16 ; 0.14]	0.00	0.02	0.844
**SR - CR**	-0.46	CoM inverted	-0.02	0.07	63.70	-0.37	[-0.17 ; 0.13]	0.00	0.02	1.000
**SL - CL**	0.46	CoM retained	-0.06	0.05	65.24	-1.19	[-0.18 ; 0.06]	0.02	0.15	0.438
**SL - CL**	0.46	CoM inverted	-0.07	0.05	63.65	-1.28	[-0.19 ; 0.05]	0.02	0.15	0.206
**SR - CR**	0.46	CoM retained	-0.08	0.07	63.09	-1.24	[-0.23 ; 0.07]	0.02	0.16	0.438
**SR - CR**	0.46	CoM inverted	-0.11	0.07	63.09	-1.69	[-0.26 ; 0.04]	0.02	0.16	0.191

Supplementary Table S 34: Type III analysis of variance table with Kenward-Roger's method of the linear mixed effects model of ∆CoP x GF/ External Torque for the geometric cues, pseudorandom condition.

	Sum Sq	Mean Sq	NumDF	DenDF	F value	Pr(>F)
**Ext. Torque**	0.450	0.450	1	1568.200	7.335	0.007
**CoM action**	8.228	8.228	1	1568.182	134.024	0.000
**Group**	0.031	0.010	3	41.982	0.169	0.917
**Ext. Torque x CoM action**	0.093	0.093	1	1568.085	1.523	0.217
**Ext. Torque x Group**	5.374	1.791	3	1568.218	29.179	0.000
**CoM action x Group**	0.139	0.046	3	1568.199	0.754	0.520
**Ext. Torque x CoM action x Group**	0.139	0.046	3	1568.087	0.754	0.520

Supplementary Table S 35: Post-hoc t-tests of pairwise comparisons based on the LMM of ∆CoP x GF/ External Torque for the geometric cues, pseudorandom condition.

contrast	external torque	CoM action	estimate	SE	df	t.ratio	conf.int	partial eta squared	Cohen's f	p
**SL - CL**	-0.46	CoM retained	0.10	0.06	62.42	1.61	[-0.04 ; 0.25]	0.04	0.20	0.224
**SL - CL**	-0.46	CoM inverted	0.03	0.06	62.10	0.45	[-0.12 ; 0.18]	0.04	0.20	1.000
**SR - CR**	-0.46	CoM retained	0.01	0.08	61.98	0.14	[-0.17 ; 0.2]	0.00	0.02	0.891
**SR - CR**	-0.46	CoM inverted	0.02	0.08	62.36	0.27	[-0.16 ; 0.21]	0.00	0.02	1.000
**SL - CL**	0.46	CoM retained	-0.09	0.06	63.80	-1.40	[-0.24 ; 0.06]	0.03	0.18	0.333
**SL - CL**	0.46	CoM inverted	-0.10	0.06	62.32	-1.49	[-0.24 ; 0.05]	0.03	0.18	0.280
**SR - CR**	0.46	CoM retained	0.00	0.08	61.79	-0.01	[-0.18 ; 0.18]	0.00	0.00	0.993
**SR - CR**	0.46	CoM inverted	-0.06	0.08	61.79	-0.70	[-0.24 ; 0.13]	0.00	0.00	0.485

Supplementary Table S 36: Type III analysis of variance table with Kenward-Roger's method of the linear mixed effects model of ∆Fy x 0.5w/ External Torque for the geometric cues, pseudorandom condition.

	Sum Sq	Mean Sq	NumDF	DenDF	F value	Pr(>F)
**Ext. Torque**	4.918	4.918	1	1568.594	129.938	0.000
**CoM action**	0.157	0.157	1	1568.540	4.150	0.042
**Group**	0.150	0.050	3	41.943	1.324	0.279
**Ext. Torque x CoM action**	0.038	0.038	1	1568.257	1.002	0.317
**Ext. Torque x Group**	0.366	0.122	3	1568.644	3.225	0.022
**CoM action x Group**	0.113	0.038	3	1568.588	0.995	0.394
**Ext. Torque x CoM action x Group**	0.041	0.014	3	1568.263	0.359	0.782

Supplementary Table S 37: Post-hoc t-tests of pairwise comparisons based on the LMM of ∆Fy x 0.5w/ External Torque for the geometric cues, pseudorandom condition.

contrast	external torque	CoM action	estimate	SE	df	t.ratio	conf.int	partial eta squared	Cohen's f	p
**SL - CL**	-0.46	CoM retained	-0.06	0.03	115.69	-1.78	[-0.14 ; 0.02]	0.03	0.17	0.154
**SL - CL**	-0.46	CoM inverted	-0.02	0.03	114.43	-0.72	[-0.1 ; 0.05]	0.03	0.17	0.579

**SR - CR**	-0.46	CoM retained	-0.02	0.04	113.93	-0.56	[-0.12 ; 0.07]	0.00	0.05	0.574
**SR - CR**	-0.46	CoM inverted	-0.04	0.04	115.44	-1.06	[-0.14 ; 0.05]	0.00	0.05	0.579
**SL - CL**	0.46	CoM retained	0.03	0.03	121.01	0.82	[-0.05 ; 0.11]	0.01	0.07	0.416
**SL - CL**	0.46	CoM inverted	0.03	0.03	115.27	0.88	[-0.05 ; 0.11]	0.01	0.07	0.404
**SR - CR**	0.46	CoM retained	-0.08	0.04	113.19	-1.91	[-0.18 ; 0.02]	0.03	0.18	0.118
**SR - CR**	0.46	CoM inverted	-0.05	0.04	113.19	-1.28	[-0.15 ; 0.04]	0.03	0.18	0.404

Supplementary Table S 38: Type III analysis of variance table with Kenward-Roger's method of the linear mixed effects model of $\Delta CoP_{lift\ off}$ of trials 4-8 in the no-cues, blocked condition

	Sum Sq	Mean Sq	NumDF	DenDF	F value	Pr(>F)
**Ext. Torque**	0.035	0.035	1	858.040	2423.892	0.000
**Group**	0.000	0.000	3	42.037	3.172	0.034
**Ext. Torque x Group**	0.000	0.000	3	858.035	4.709	0.003

Supplementary Table S 39: Post-hoc t-tests of pairwise comparisons between stroke patient- and hand-matched control groups based on the marginal means of the LMM of $\Delta CoP_{lift\ off}$ of trials 4-8 in the no-cues, blocked condition with Holm-Bonferroni correction for multiple testing applied

contrast	external torque	estimate	SE	df	t.ratio	conf.int	partial eta squared	Cohen's f	p
**SL - CL**	-0.21	0	0	46.04	-2.10	[-0.01 ; 0]	0.09	0.31	0.083
**SR - CR**	-0.21	0	0	46.44	1.40	[0 ; 0.01]	0.04	0.21	0.168
**SL - CL**	0.21	0	0	46.04	-2.38	[-0.01 ; 0]	0.11	0.35	0.043
**SR - CR**	0.21	0	0	46.49	-0.20	[0 ; 0]	0.00	0.03	0.844

Supplementary Table S 40: Type III analysis of variance table with Kenward-Roger's method of the linear mixed effects model of $\Delta CoP_{lift\ off}$ for the no-cues, pseudorandom condition.

	Sum Sq	Mean Sq	NumDF	DenDF	F value	Pr(>F)
**Ext. Torque**	0.001	0.001	1	1568.061	117.156	0.000
**CoM action**	0.000	0.000	1	1568.223	0.299	0.585
**Group**	0.000	0.000	3	42.027	0.860	0.469
**Ext. Torque x CoM action**	0.008	0.008	1	1568.067	682.310	0.000
**Ext. Torque x Group**	0.000	0.000	3	1568.054	0.593	0.620
**CoM action x Group**	0.000	0.000	3	1568.192	0.529	0.662
**Ext. Torque x CoM action x Group**	0.000	0.000	3	1568.059	1.888	0.130

Supplementary Table S 41: Post-hoc t-tests of pairwise comparisons based on the LMM of $\Delta CoP_{lift\ off}$ for the no-cues, pseudorandom condition.

contrast	external torque	CoM action	estimate	SE	df	t.ratio	conf.int	partial eta squared	Cohen's f	p
**SL - CL**	-0.21	CoM retained	0	0	50.16	-1.35	[0 ; 0]	0.03	0.19	0.368
**SL - CL**	-0.21	CoM inverted	0	0	50.16	-1.18	[0 ; 0]	0.03	0.19	0.489
**SR - CR**	-0.21	CoM retained	0	0	50.59	-0.80	[-0.01 ; 0]	0.01	0.11	0.425
**SR - CR**	-0.21	CoM inverted	0	0	50.78	-1.12	[-0.01 ; 0]	0.01	0.11	0.489
**SL - CL**	0.21	CoM retained	0	0	50.20	-0.95	[0 ; 0]	0.02	0.13	0.607
**SL - CL**	0.21	CoM inverted	0	0	50.28	-1.53	[0 ; 0]	0.02	0.13	0.266
**SR - CR**	0.21	CoM retained	0	0	50.59	-1.04	[-0.01 ; 0]	0.02	0.15	0.607
**SR - CR**	0.21	CoM inverted	0	0	51.05	0.03	[0 ; 0]	0.02	0.15	0.975

Supplementary Table S 42: Type III analysis of variance table with Kenward-Roger's method of the linear mixed effects model of $\Delta CoP_{lift\ off}$ of trials 4-8 in the geometric cues, blocked condition.

	Sum Sq	Mean Sq	NumDF	DenDF	F value	Pr(>F)
**Ext. Torque**	0.172	0.172	1	859.321	4293.409	0.000
**Group**	0.000	0.000	3	42.011	4.111	0.012
**Ext. Torque x Group**	0.000	0.000	3	859.306	4.074	0.007

Supplementary Table S 43: Post-hoc t-tests of pairwise comparisons based on the LMM of $\Delta CoP_{lift\ off}$ of trials 4-8 in the geometric cues, blocked condition.

contrast	external torque	estimate	SE	df	t.ratio	conf.int	partial eta squared	Cohen's f	p
**SL - CL**	-0.46	0	0	54.73	-0.94	[0 ; 0]	0.02	0.13	0.527
**SR - CR**	-0.46	0	0	55.13	-1.13	[-0.01 ; 0]	0.02	0.15	0.527
**SL - CL**	0.46	0	0	55.26	-1.70	[-0.01 ; 0]	0.05	0.23	0.189
**SR - CR**	0.46	0	0	54.97	-0.36	[-0.01 ; 0]	0.00	0.05	0.717

Supplementary Table S 44: Type III analysis of variance table with Kenward-Roger's method of the linear mixed effects model of $\Delta CoP_{lift\ off}$ for the geometric cues, pseudorandom condition.

	Sum Sq	Mean Sq	NumDF	DenDF	F value	Pr(>F)
**Ext. Torque**	0.198	0.198	1	1568.216	5620.874	0.000
**CoM action**	0.000	0.000	1	1568.196	0.600	0.439
**Group**	0.000	0.000	3	41.980	1.058	0.377
**Ext. Torque x CoM action**	0.002	0.002	1	1568.092	66.302	0.000
**Ext. Torque x Group**	0.001	0.000	3	1568.235	5.276	0.001
**CoM action x Group**	0.000	0.000	3	1568.214	0.385	0.764
**Ext. Torque x CoM action x Group**	0.000	0.000	3	1568.094	0.107	0.956

Supplementary Table S 45: Post-hoc t-tests of pairwise comparisons based on the LMM of $\Delta CoP_{lift\ off}$ for the geometric cues, pseudorandom condition.

contrast	external torque	CoM action	estimate	SE	df	t.ratio	conf.int	partial eta squared	Cohen's f	p
**SL - CL**	-0.46	CoM retained	0	0	64.21	-0.53	[0 ; 0]	0.00	0.07	0.595
**SL - CL**	-0.46	CoM inverted	0	0	63.86	0.16	[0 ; 0]	0.00	0.07	0.875
**SR - CR**	-0.46	CoM retained	0	0	63.73	-1.09	[-0.01 ; 0]	0.02	0.14	0.556
**SR - CR**	-0.46	CoM inverted	0	0	64.15	-1.04	[-0.01 ; 0]	0.02	0.14	0.602
**SL - CL**	0.46	CoM retained	0	0	65.72	-1.67	[-0.01 ; 0]	0.04	0.21	0.200
**SL - CL**	0.46	CoM inverted	0	0	64.10	-1.35	[-0.01 ; 0]	0.04	0.21	0.366
**SR - CR**	0.46	CoM retained	0	0	63.52	-0.57	[-0.01 ; 0]	0.01	0.07	0.568
**SR - CR**	0.46	CoM inverted	0	0	63.52	-0.96	[-0.01 ; 0]	0.01	0.07	0.366

Supplementary Table S 46: Type III analysis of variance table with Kenward-Roger's method of the linear mixed effects model of $GF_{lift\ off}$ of trials 4-8 in the no-cues, blocked condition

	Sum Sq	Mean Sq	NumDF	DenDF	F value	Pr(>F)
**Ext. Torque**	55.822	55.822	1	858.010	5.211	0.023
**Group**	2.671	0.890	3	42.010	0.083	0.969
**Ext. Torque x Group**	61.327	20.442	3	858.008	1.908	0.127

Supplementary Table S 47: Post-hoc t-tests of pairwise comparisons between stroke patient- and hand-matched control groups based on the marginal means of the LMM of

$GF_{lift\ off}$ of trials 4-8 in the no-cues, blocked condition with Holm-Bonferroni correction for multiple testing applied.

contrast	external torque	estimate	SE	df	t.ratio	conf.int	partial eta squared	Cohen's f	p
SL - CL	-0.21	-0.44	2.61	42.94	-0.17	[-6.51 ; 5.64]	0	0.03	1
SR - CR	-0.21	0.68	3.25	43.03	0.21	[-6.87 ; 8.24]	0	0.03	1
SL - CL	0.21	0.89	2.61	42.94	0.34	[-5.19 ; 6.96]	0	0.05	1
SR - CR	0.21	0.61	3.25	43.04	0.19	[-6.95 ; 8.17]	0	0.03	1

Supplementary Table S 48: Type III analysis of variance table with Kenward-Roger's method of the linear mixed effects model of $GF_{lift\ off}$ for the no-cues, pseudorandom condition.

	Sum Sq	Mean Sq	NumDF	DenDF	F value	Pr(>F)
Ext. Torque	0.092	0.092	1	1568.018	0.006	0.939
CoM action	196.395	196.395	1	1568.068	12.445	0.000
Group	2.276	0.759	3	42.009	0.048	0.986
Ext. Torque x CoM action	12.361	12.361	1	1568.020	0.783	0.376
Ext. Torque x Group	72.113	24.038	3	1568.016	1.523	0.207
CoM action x Group	129.315	43.105	3	1568.058	2.732	0.043
Ext. Torque x CoM action x Group	45.023	15.008	3	1568.018	0.951	0.415

Supplementary Table S 49: Post-hoc t-tests of pairwise comparisons based on the LMM of $GF_{lift\ off}$ for the no-cues, pseudorandom condition.

contrast	external torque	CoM action	estimate	SE	df	t.ratio	conf.int	partial eta squared	Cohen's f	p
SL - CL	-0.21	CoM retained	-1.11	2.66	44.31	-0.42	[-7.28 ; 5.06]	0	0.06	1
SL - CL	-0.21	CoM inverted	0.12	2.66	44.31	0.05	[-6.04 ; 6.29]	0	0.06	1
SR - CR	-0.21	CoM retained	0.30	3.31	44.43	0.09	[-7.37 ; 7.98]	0	0.01	1
SR - CR	-0.21	CoM inverted	1.22	3.31	44.48	0.37	[-6.45 ; 8.9]	0	0.01	1
SL - CL	0.21	CoM retained	-0.57	2.66	44.32	-0.21	[-6.74 ; 5.6]	0	0.03	1
SL - CL	0.21	CoM inverted	-0.39	2.66	44.35	-0.15	[-6.56 ; 5.77]	0	0.03	1
SR - CR	0.21	CoM retained	-0.32	3.31	44.43	-0.10	[-8 ; 7.35]	0	0.01	1
SR - CR	0.21	CoM inverted	-0.36	3.31	44.56	-0.11	[-8.04 ; 7.32]	0	0.01	1

Supplementary Table S 50: Type III analysis of variance table with Kenward-Roger's method of the linear mixed effects model of $GF_{lift\ off}$ of trials 4-8 in the geometric cues, blocked condition.

	Sum Sq	Mean Sq	NumDF	DenDF	F value	Pr(>F)
Ext. Torque	28.273	28.273	1	859.042	1.841	0.175
Group	12.643	4.214	3	42.002	0.274	0.844
Ext. Torque x Group	404.409	134.803	3	859.040	8.776	0.000

Supplementary Table S 51: Post-hoc t-tests of pairwise comparisons based on the LMM of $GF_{lift\ off}$ of trials 4-8 in the geometric cues, blocked condition.

contrast	external torque	estimate	SE	df	t.ratio	conf.int	partial eta squared	Cohen's f	p
SL - CL	-0.46	0.83	2.47	43.54	0.34	[-4.91 ; 6.58]	0.00	0.05	1.000
SR - CR	-0.46	0.50	3.08	43.59	0.16	[-6.65 ; 7.65]	0.00	0.02	1.000
SL - CL	0.46	3.06	2.48	43.61	1.24	[-2.69 ; 8.8]	0.03	0.19	0.447
SR - CR	0.46	0.34	3.08	43.57	0.11	[-6.81 ; 7.49]	0.00	0.02	0.913

Supplementary Table S 52: Type III analysis of variance table with Kenward-Roger's method of the linear mixed effects model of $GF_{lift\ off}$ for the geometric cues, pseudorandom condition.

	Sum Sq	Mean Sq	NumDF	DenDF	F value	Pr(>F)
**Ext. Torque**	69.228	69.228	1	1568.031	4.410	0.036
**CoM action**	220.841	220.841	1	1568.028	14.068	0.000
**Group**	10.525	3.508	3	41.997	0.223	0.880
**Ext. Torque x CoM action**	25.503	25.503	1	1568.013	1.625	0.203
**Ext. Torque x Group**	103.032	34.344	3	1568.034	2.188	0.088
**CoM action x Group**	64.954	21.651	3	1568.031	1.379	0.247
**Ext. Torque x CoM action x Group**	28.986	9.662	3	1568.013	0.615	0.605

Supplementary Table S 53: Post-hoc t-tests of pairwise comparisons based on the LMM of $GF_{lift\ off}$ for the geometric cues, pseudorandom condition.

contrast	external torque	CoM action	estimate	SE	df	t.ratio	conf.int	partial eta squared	Cohen's f	p
**SL - CL**	-0.46	CoM retained	1.61	2.43	44.87	0.66	[-4.03 ; 7.24]	0.01	0.10	1.000
**SL - CL**	-0.46	CoM inverted	1.38	2.43	44.83	0.57	[-4.25 ; 7.02]	0.01	0.10	1.000
**SR - CR**	-0.46	CoM retained	0.29	3.02	44.81	0.10	[-6.72 ; 7.3]	0.00	0.01	1.000
**SR - CR**	-0.46	CoM inverted	1.81	3.02	44.86	0.60	[-5.2 ; 8.82]	0.00	0.01	1.000
**SL - CL**	0.46	CoM retained	2.40	2.43	45.05	0.99	[-3.24 ; 8.04]	0.02	0.15	0.657
**SL - CL**	0.46	CoM inverted	2.09	2.43	44.85	0.86	[-3.54 ; 7.73]	0.02	0.15	0.787
**SR - CR**	0.46	CoM retained	0.41	3.02	44.78	0.13	[-6.6 ; 7.42]	0.00	0.02	0.894
**SR - CR**	0.46	CoM inverted	0.23	3.02	44.78	0.08	[-6.78 ; 7.24]	0.00	0.02	0.939
